# Functional and Therapeutic Roles of Plant-Derived Antioxidants in Type 2 Diabetes Mellitus: Mechanisms, Challenges, and Considerations for Special Populations

**DOI:** 10.3390/antiox14060725

**Published:** 2025-06-13

**Authors:** Vicente Javier Clemente-Suárez, Alexandra Martín-Rodríguez, Ana Isabel Beltrán-Velasco, Alejandro Rubio-Zarapuz, Ismael Martínez-Guardado, Roberto Valcárcel-Martín, José Francisco Tornero-Aguilera

**Affiliations:** 1 Faculty of Medicine, Health and Sports, Universidad Europea de Madrid, Villaviciosa de Odón, 28670 Madrid, Spain; vctxente@yahoo.es (V.J.C.-S.); alejandro.rubio@universidadeuropea.es (A.R.-Z.); 2 Faculty of Health Sciences, UNIE University, 28015 Madrid, Spain; roberto.valcarcel@universidadunie.com; 3 Psychology Department, Faculty of Life and Natural Sciences, Nebrija University, 28240 Madrid, Spain; abeltranv@nebrija.es; 4 LFE Research Group, Department of Health and Human Performance, Faculty of Physical Activity and SportScience (INEF), Universidad Politécnica de Madrid, Calle de Martín Fierro, 7, 28040 Madrid, Spain; imartinezgu91@gmail.com; 5 Kos Generating Health, 45007 Toledo, Spain; doctorneroaguilera@gmail.com

**Keywords:** type 2 diabetes mellitus, antioxidants, oxidative stress, insulin resistance, polyphenols

## Abstract

Background: Type 2 diabetes mellitus (T2DM) is a chronic metabolic disorder characterized by persistent hyperglycemia, oxidative stress, and inflammation, contributing to insulin resistance and long-term complications. Dietary antioxidants from plant sources, such as polyphenols, flavonoids, carotenoids, and phenolic acids, have been increasingly studied for their potential to modulate these pathophysiological mechanisms. Objective: This review aims to summarize and critically analyze the current evidence on the biological effects, therapeutic potential, and translational challenges of plant-derived antioxidants in the prevention and management of T2DM. Methods: This narrative review was conducted using peer-reviewed literature from PubMed, Scopus, and Web of Science. Emphasis was placed on mechanistic studies, clinical trials, bioavailability data, and advances in formulation technologies related to antioxidant compounds in the context of T2DM. Results: Plant antioxidants exert beneficial effects by modulating oxidative stress, reducing systemic inflammation, and improving insulin signaling pathways. However, their clinical application is limited by low bioavailability, chemical instability, and high interindividual variability. Recent developments, such as nanoencapsulation, synergistic functional food formulations, and microbiome-targeted strategies, have shown promise in enhancing efficacy. Additionally, personalized nutrition approaches and regulatory advances are emerging to support the integration of antioxidant-based interventions into diabetes care. Conclusions: Plant-derived antioxidants represent a promising complementary tool for T2DM management. Nonetheless, their effective clinical use depends on overcoming pharmacokinetic limitations and validating their long-term efficacy in well-designed trials. Integrating food technology, microbiome science, and precision nutrition will be crucial to translate these compounds into safe, scalable, and personalized therapeutic options for individuals with or at risk of T2DM.

## 1. Introduction

Type 2 diabetes mellitus (T2DM) is a rapidly escalating global health concern currently affecting over 537 million individuals worldwide and projected to reach 783 million by 2045 [1]. Characterized by chronic hyperglycemia and insulin resistance, T2DM is closely associated with increased oxidative stress and systemic inflammation [2], which play pivotal roles in its pathogenesis and complications. Reactive oxygen species (ROS) disrupt insulin signaling pathways and promote β-cell dysfunction, while pro-inflammatory cytokines further impair glucose homeostasis, establishing a vicious cycle that accelerates disease progression [3]. In this context, dietary strategies targeting redox balance and inflammatory processes have garnered substantial interest. Among them, plant-derived antioxidants, such as polyphenols, flavonoids, and carotenoids, have emerged as promising therapeutic agents due to their multifaceted bioactivity and natural origin [4,5]. These compounds exert their protective effects not only by scavenging ROS but also by modulating key molecular pathways, including AMP-activated protein kinase (AMPK), nuclear factor kappa B (NF-κB), and peroxisome proliferator-activated receptors (PPARs), which are essential regulators of metabolic homeostasis [6,7].

### Methodology

To ensure the scientific rigor, relevance, and clarity of this narrative review, the following methodological steps were followed:

- Structured literature search: Conducted across five major scientific databases—PubMed, Scopus, Web of Science, Embase, and ScienceDirect—to ensure broad and multidisciplinary coverage.
- Timeframe and study types: Included peer-reviewed original research articles and systematic reviews published between January 2010 and March 2024.
- Quality prioritization: Emphasis was placed on studies published in high-impact journals, particularly those indexed in Journal Citation Reports (JCR) and SCImago Journal Rank (SJR), with a focus on Q1 journals in the fields of endocrinology, pharmacology, and nutrition.
- Search strategy: Designed to reflect the multifactorial nature of type 2 diabetes mellitus (T2DM) and its interplay with oxidative stress and plant-based interventions.
  Keywords and Boolean operators included “type 2 diabetes mellitus”, “oxidative stress”, “plant-derived antioxidants”, “polyphenols”, “flavonoids”, “carotenoids”, “insulin resistance”, “AMP-activated protein kinase (AMPK)”, “NF-κB”, “gut microbiota”, “inflammation”, and “nutrigenomics.”
- Inclusion criteria:
  Experimental and clinical studies involving in vitro, in vivo, or human subjects.
- Exclusion criteria:
  Publications not in English.
  Studies lacking experimental or clinical validation.
  Narrative commentaries, dissertations, books, conference abstracts, or preprints.
  Articles considered methodologically outdated or not aligned with current pathophysiological understanding.
- Scope of the review:
  To critically synthesize current evidence on the mechanistic and translational role of plant-derived antioxidants in T2DM.
  Special focus on their ability to mitigate oxidative stress, modulate inflammation, and improve insulin signaling.
  Highlight key limitations (e.g., bioavailability, metabolic stability) and emerging research directions, including microbiome interactions, synergistic antioxidant strategies, and personalized nutrition.

This narrative review critically synthesizes evidence from molecular, nutritional, and clinical research on the potential of plant-derived antioxidants to modulate oxidative stress, inflammation, and insulin resistance in T2DM. It also discusses key challenges, such as bioavailability and metabolic stability, and explores emerging strategies, including synergistic formulations, microbiome modulation, and precision nutrition.

## 2. Oxidative Stress, Inflammation, and Insulin Resistance in Type 2 Diabetes Mellitus

T2DM is a complex metabolic disorder characterized by chronic hyperglycemia, insulin resistance, and β-cell dysfunction. One of the central pathological mechanisms underlying T2DM is oxidative stress, which results from an imbalance between reactive oxygen species (ROS) production and the antioxidant defense system [8]. Elevated ROS levels contribute to mitochondrial dysfunction, endoplasmic reticulum stress, and β-cell apoptosis, exacerbating insulin resistance and impairing glucose homeostasis [9]. In addition, oxidative stress triggers a cascade of inflammatory responses, which further deteriorate insulin signaling pathways and worsen metabolic dysfunction [10]. Chronic low-grade inflammation is another hallmark of T2DM and is closely linked to oxidative stress. Pro-inflammatory cytokines, such as tumor necrosis factor-alpha (TNF-α), interleukin-6 (IL-6), and interleukin-1β (IL-1β), are upregulated in individuals with diabetes, contributing to insulin receptor desensitization and impaired glucose uptake [11]. These cytokines activate nuclear factor kappa B (NF-κB) and c-Jun N-terminal kinase (JNK) signaling pathways, which directly interfere with insulin receptor substrate (IRS) phosphorylation, leading to insulin resistance [12]. Additionally, the accumulation of advanced glycation end-products (AGEs) in diabetic patients exacerbates oxidative stress and inflammatory signaling, further impairing metabolic control [13].

The interplay between oxidative stress and inflammation is particularly detrimental in the adipose tissue of individuals with obesity-related T2DM. Dysfunctional adipocytes release excessive free fatty acids (FFAs), which not only serve as substrates for oxidative stress but also activate Toll-like receptors (TLRs), perpetuating inflammatory cascades [14]. This pro-inflammatory and oxidative environment disrupts insulin signaling and fosters ectopic lipid accumulation in the liver and the skeletal muscle, further aggravating insulin resistance [15]. Consequently, targeting oxidative stress and inflammation has emerged as a crucial therapeutic approach in the management of T2DM.

Given the central role of oxidative stress in T2DM’s pathogenesis, endogenous antioxidant systems, such as superoxide dismutase (SOD), catalase (CAT), and glutathione peroxidase (GPx), are often overwhelmed in diabetic patients [16]. The depletion of these enzymatic antioxidants results in an unchecked accumulation of ROS, further impairing insulin sensitivity and β-cell function. This has led to an increasing interest in dietary and pharmacological antioxidants as potential interventions to restore redox homeostasis and mitigate metabolic dysfunction in T2DM [17].

Plant-derived antioxidants, including polyphenols, flavonoids, and carotenoids, have gained considerable attention for their ability to counteract oxidative stress and improve insulin sensitivity [4]. These bioactive compounds exert their protective effects through multiple mechanisms, including scavenging ROS, modulating inflammatory pathways, and enhancing mitochondrial function [5]. Furthermore, some plant antioxidants directly target key molecular regulators of insulin signaling, such as AMP-activated protein kinase (AMPK) and peroxisome proliferator-activated receptor gamma (PPAR-γ), making them promising candidates for T2DM management [7].

Recent clinical and preclinical studies have demonstrated that diets rich in plant-based antioxidants correlate with improved glycemic control, reduced oxidative damage, and lower inflammatory markers in diabetic patients [18]. However, challenges related to the bioavailability, metabolism, and stability of these compounds remain key hurdles in their clinical translation [19]. Addressing these limitations through advanced formulation strategies and nutrigenomic approaches could pave the way for more effective personalized antioxidant therapies for T2DM [14].

The interplay between these mechanisms contributes to progressive β-cell dysfunction and metabolic deterioration. Studies have demonstrated that oxidative stress induced by excessive ROS production disrupts insulin signaling pathways and induces chronic inflammation, creating a vicious cycle that perpetuates metabolic disturbances [20].

Mitochondria are central regulators of cellular energy metabolism, and their dysfunction is a hallmark of insulin resistance. In T2DM, excessive nutrient influx—particularly from high-fat and high-glucose diets—leads to mitochondrial overload and excessive ROS generation [21]. This oxidative burden impairs mitochondrial dynamics, reducing ATP synthesis and leading to structural damage in key insulin-responsive tissues, such as skeletal muscle, liver, and adipose tissue [22].

Mitochondrial ROS also contribute to defective insulin signaling by activating stress kinases, such as JNK and inhibitor of nuclear factor kappa-B kinase subunit beta (IKKβ), both of which inhibit insulin receptor substrate (IRS) phosphorylation, ultimately impairing glucose uptake [23]. Furthermore, oxidative damage to mitochondrial DNA (mtDNA) exacerbates cellular stress and promotes further metabolic dysregulation [24]. Strategies aimed at enhancing mitochondrial function, such as antioxidant therapies, caloric restriction, and physical activity, have been proposed as potential interventions to mitigate insulin resistance [25].

Beyond mitochondria, the endoplasmic reticulum (ER) also plays a critical role in oxidative-stress-induced insulin resistance. The ER is responsible for protein folding and secretion, particularly of insulin in pancreatic β-cells. However, in the hyperglycemic and hyperlipidemic state of T2DM, ER stress is triggered due to protein misfolding, leading to activation of the unfolded protein response (UPR) [26]. Prolonged UPR activation results in apoptosis of β-cells through the PERK-eIF2α-CHOP pathway, contributing to reduced insulin secretion and worsening hyperglycemia [27]. The interplay between ER stress and oxidative stress is bidirectional; ROS can exacerbate ER stress, and ER stress can further promote oxidative damage via calcium dysregulation and activation of NADPH oxidase [28]. Recent studies suggest that pharmacological agents targeting ER stress pathways, such as tauroursodeoxycholic acid (TUDCA), may help improve insulin sensitivity and β-cell survival in T2DM [29,30].

Chronic, low-grade inflammation is a defining feature of T2DM largely driven by adipose tissue dysfunction. In individuals with obesity-related insulin resistance, hypertrophied adipocytes exhibit increased secretion of pro-inflammatory cytokines, such as TNF-α, IL-6, and IL-1β, all of which impair insulin signaling by activating NF-κB and JNK pathways [31]. Additionally, macrophage infiltration into adipose tissue shifts the immune balance toward a pro-inflammatory M1 phenotype, further exacerbating metabolic inflammation [31]. The role of inflammation in insulin resistance extends beyond adipose tissue to the liver and muscle, where increased cytokine signaling contributes to hepatic gluconeogenesis and reduced glucose uptake [32]. Emerging research highlights the importance of resolving inflammation as a potential therapeutic target for T2DM, with interventions like omega-3 fatty acids, flavonoids, and anti-inflammatory drugs demonstrating promising effects on glycemic control.

Recent evidence suggests that the gut microbiota plays a crucial role in modulating oxidative stress and inflammation in T2DM. Dysbiosis, characterized by an imbalance in gut microbial composition, has been associated with increased intestinal permeability, leading to systemic inflammation via endotoxin release [33]. Lipopolysaccharides (LPS) derived from Gram-negative bacteria activate TLR4 signaling, further amplifying inflammatory responses and promoting insulin resistance [34]. Certain dietary antioxidants, such as polyphenols and prebiotics, have been shown to modulate gut microbiota composition, increasing beneficial bacteria while reducing inflammation-associated taxa [35]. This suggests that gut-microbiota-targeted therapies could serve as a novel approach to managing oxidative stress and metabolic dysfunction in T2DM.

Given the intricate relationship between oxidative stress, inflammation, and insulin resistance, targeting these pathways offers a promising avenue for T2DM treatment. Current therapeutic approaches focus on lifestyle modifications, such as dietary interventions rich in antioxidants and anti-inflammatory compounds, regular physical activity, and weight management [20]. Pharmacological strategies, including metformin, SGLT2 inhibitors, and GLP-1 receptor agonists, also exert beneficial effects by reducing oxidative and inflammatory burden in T2DM patients [2].

## 3. Classification and Bioactivity of Plant-Derived Antioxidants

Plant-derived antioxidants represent a diverse group of bioactive compounds that are integral to the neutralization of reactive oxygen species (ROS) and the mitigation of oxidative stress, a pathological hallmark of numerous chronic diseases, including type 2 diabetes mellitus (T2DM). Oxidative stress arises from an imbalance between the production of ROS and endogenous antioxidant defense mechanisms, leading to cellular and molecular damage that exacerbates insulin resistance, β-cell dysfunction, and systemic inflammation [36]. In T2DM, the overproduction of ROS is closely linked to hyperglycemia-induced mitochondrial dysfunction, advanced glycation end-products (AGEs) formation, and the activation of pro-inflammatory pathways, all of which contribute to the progression of diabetic complications [37].

Plant-derived antioxidants, which include polyphenols, carotenoids, vitamins, and organosulfur compounds, exhibit a wide range of chemical structures and biological activities. These compounds not only directly scavenge ROS but also modulate key signaling pathways involved in oxidative stress, inflammation, and glucose homeostasis [38]. For instance, polyphenols, such as flavonoids and phenolic acids, have been shown to enhance insulin sensitivity by activating AMP-activated protein kinase (AMPK) and peroxisome proliferator-activated receptor gamma (PPAR-γ) pathways, while carotenoids like β-carotene and lutein protect against oxidative damage by quenching singlet oxygen and stabilizing cell membranes [39]. Furthermore, the bioactivity of these antioxidants is influenced by their bioavailability, stability, and interactions with the gut microbiota, which can modify their metabolic fate and therapeutic efficacy [40]. Furthermore, the growing interest in plant-derived antioxidants stems from their potential to serve as complementary or alternative therapeutic agents in the management of T2DM. Unlike synthetic antioxidants, which may have limited efficacy and potential side effects, natural antioxidants offer a safer and more sustainable approach to reducing oxidative stress and improving metabolic health [41]. Moreover, the synergistic effects of antioxidant combinations, as well as advancements in nutrigenomics and personalized nutrition, have opened new avenues for optimizing their use in diabetes prevention and treatment.

### 3.1. Classification of Plant-Derived Antioxidants

#### 3.1.1. Polyphenols

Polyphenols are a structurally diverse and biologically active class of secondary metabolites widely distributed in the plant kingdom characterized by multiple phenolic rings and hydroxyl groups that confer significant antioxidant properties. Their capacity to donate hydrogen atoms or electrons, chelate transition metals, and modulate intracellular signaling pathways underpins their protective effects against oxidative stress and inflammation [42]. Polyphenols have been extensively studied for their role in mitigating oxidative damage, modulating the gut microbiota, and influencing metabolic pathways involved in chronic diseases, such as type 2 diabetes mellitus (T2DM) and cardiovascular disorders [43].

##### Flavonoids

Flavonoids constitute the largest and most studied subclass of polyphenols, comprising over 6000 bioactive compounds categorized into flavonols, flavones, flavanones, flavanols (catechins), anthocyanins, and isoflavones. These compounds exhibit strong free radical scavenging properties, interact with key enzymes in cellular metabolism, and modulate gene expression related to antioxidant defenses [44]. Their distribution in dietary sources, such as fruits, vegetables, tea, cocoa, and wine, correlates with their health-promoting effects.

- Quercetin: A predominant flavonol found in apples, onions, and berries, quercetin exerts multifaceted biological effects, including the regulation of glucose homeostasis through the activation of AMP-activated protein kinase (AMPK) and the facilitation of glucose transporter type 4 (GLUT4) translocation in skeletal muscle cells. Furthermore, it downregulates hepatic gluconeogenesis by inhibiting the expression of phosphoenolpyruvate carboxykinase (PEPCK) and glucose-6-phosphatase (G6Pase), enzymes critical for glucose production [45].
- Kaempferol, also a flavonol found in leafy greens and berries, has demonstrated glucose-lowering effects and mitochondrial protective properties through the activation of PGC-1α and the inhibition of JNK phosphorylation.
- Catechins: Predominantly found in green tea, catechins, particularly epigallocatechin gallate (EGCG), have been demonstrated to exert significant antioxidant and anti-inflammatory properties. EGCG enhances insulin sensitivity by modulating the insulin receptor substrate (IRS)/phosphatidylinositol 3-kinase (PI3K)/Akt pathway, thereby improving glucose uptake and metabolic regulation. Additionally, catechins exhibit neuroprotective properties by modulating oxidative-stress-related pathways in neurodegenerative disorders [46].
- Anthocyanins: These pigmented flavonoids, found in berries, red grapes, and purple corn, exhibit strong antioxidant and anti-inflammatory activities. Studies have demonstrated their capacity to enhance insulin secretion from pancreatic β-cells, reduce postprandial hyperglycemia, and inhibit the activation of nuclear factor-kappa B (NF-κB), a key regulator of inflammatory responses. Moreover, anthocyanins have been implicated in modulating gut microbiota composition, fostering the proliferation of beneficial bacterial species while inhibiting pathogenic strains, thus exerting systemic metabolic benefits [47].
- Isoflavones, such as genistein from soy, have estrogen-like activity and improve insulin’s action by interacting with PPARγ and reducing oxidative damage [48].

##### Phenolic Acids

Phenolic acids constitute another major subgroup of polyphenols, with hydroxycinnamic acids (e.g., ferulic acid, caffeic acid) and hydroxybenzoic acids (e.g., gallic acid, protocatechuic acid) being the most prevalent forms. These compounds are commonly found in whole grains, coffee, fruits, and vegetables and possess potent antioxidant, anti-inflammatory, and anti-diabetic properties [49].

- Ferulic acid: Predominantly present in rice bran, oats, and wheat, ferulic acid exerts its antioxidant effects by scavenging reactive oxygen species (ROS) and enhancing the activity of endogenous antioxidant enzymes, such as superoxide dismutase (SOD) and catalase (CAT). Furthermore, it has been shown to modulate nitric oxide (NO) bioavailability, improving endothelial function and vascular health in metabolic disorders [50].
- Caffeic acid: Commonly found in coffee, fruits, and herbs, caffeic acid exhibits strong anti-inflammatory properties by inhibiting the production of pro-inflammatory cytokines, such as tumor necrosis factor-alpha (TNF-α) and interleukin-6 (IL-6), thereby modulating systemic inflammatory responses. Moreover, it has been shown to attenuate lipid peroxidation and oxidative damage in neuronal cells, suggesting potential neuroprotective effects [51].
- Hydroxybenzoic acids, such as gallic acid and protocatechuic acid, are abundant in berries, tea, and wine. Gallic acid has been shown to protect β-cells from oxidative injury, reduce hepatic gluconeogenesis, and attenuate pro-inflammatory cytokine production by modulating MAPK and NF-κB signaling. Protocatechuic acid has been linked to improved lipid metabolism and reduced insulin resistance in animal models [52].
- Resveratrol: A naturally occurring stilbene found in grapes, red wine, and peanuts, resveratrol has garnered significant attention due to its ability to activate sirtuin 1 (SIRT1), a protein deacetylase implicated in mitochondrial function, insulin sensitivity, and longevity. Its cardioprotective effects are mediated through the enhancement of endothelial nitric oxide synthase (eNOS) activity, the reduction of oxidative stress, and the attenuation of inflammatory cascades [53].
- Lignans: These phytoestrogenic compounds, primarily found in flaxseeds and sesame seeds, exhibit antioxidative and lipid-lowering effects. Secoisolariciresinol diglucoside (SDG), a major lignan, has been shown to modulate gut microbiota metabolism, enhance short-chain fatty acid production, and reduce systemic oxidative stress markers in diabetic individuals [54].

#### 3.1.2. Carotenoids

Carotenoids are a class of lipophilic antioxidants predominantly found in pigmented fruits and vegetables, such as carrots, tomatoes, and spinach. These compounds are classified into carotenes (e.g., β-carotene) and xanthophylls (e.g., lutein and zeaxanthin). Their antioxidant activity is primarily mediated through singlet oxygen quenching and peroxyl radical scavenging, mechanisms that confer photoprotective and anti-inflammatory effects [55].

- β-Carotene: A provitamin A carotenoid abundant in carrots, sweet potatoes, and leafy greens, β-carotene exerts significant antioxidant effects by neutralizing reactive oxygen species (ROS), particularly singlet oxygen and lipid peroxyl radicals. In experimental models of T2DM, β-carotene supplementation has been shown to reduce oxidative stress markers, such as malondialdehyde (MDA), increase antioxidant enzyme activities, including glutathione peroxidase (GPx) and catalase (CAT), and enhance insulin sensitivity. Furthermore, β-carotene has been implicated in the inhibition of the formation of advanced glycation end-products (AGEs), which are linked to diabetic vascular complications. Comparative studies suggest that β-carotene may act synergistically with vitamin E in preserving membrane integrity and suppressing pro-inflammatory cytokine release, particularly in the context of high-fat-diet-induced insulin resistance [56].
- Lutein and zeaxanthin: These xanthophylls, found in green leafy vegetables, corn, and egg yolks, have been extensively studied for their protective effects on retinal health, especially in diabetic retinopathy. Mechanistically, both compounds reduce ROS accumulation in retinal pigment epithelial cells and inhibit nuclear factor-kappa B (NF-κB) signaling, leading to the decreased expression of pro-inflammatory mediators, such as TNF-α and IL-6. Beyond their ocular benefits, systemic administration of lutein in diabetic rodents has been associated with improved lipid metabolism, increased adiponectin levels, and the attenuation of hepatic steatosis. Clinical studies also report reductions in circulating C-reactive protein (CRP) and improvements in antioxidant capacity following lutein supplementation in patients with metabolic syndrome and T2DM [57].

Notably, their absorption and bioavailability are enhanced when consumed with dietary fats, highlighting the importance of food matrix considerations in therapeutic design. Despite their promising roles, the clinical translation of carotenoids is limited by variable bioavailability, susceptibility to oxidation, and differences in individual absorption kinetics. Recent advances in delivery systems, such as nanoemulsions and liposomal encapsulation, have been proposed to enhance the stability and gastrointestinal uptake of carotenoids. Additionally, emerging evidence suggests that their metabolic activity may be partly mediated through modulation of gut microbiota and bile acid metabolism, offering new avenues for targeted interventions in metabolic disorders.

#### 3.1.3. Vitamins

Vitamins with antioxidant properties, particularly vitamin C (ascorbic acid) and vitamin E (tocopherols and tocotrienols), play essential roles in modulating oxidative stress, inflammation, and insulin signaling pathways relevant to T2DM. These micronutrients act not only as direct scavengers of ROS but also as synergistic agents within the endogenous antioxidant network, influencing redox-sensitive signaling and cellular homeostasis.

- Vitamin C: As a water-soluble antioxidant, vitamin C acts by directly neutralizing a broad spectrum of ROS, including superoxide anion, hydroxyl radicals, and singlet oxygen. Importantly, it also regenerates oxidized vitamin E, thus maintaining the redox cycle between aqueous and lipid compartments. In patients with T2DM, vitamin C supplementation has been shown to reduce plasma levels of malondialdehyde (MDA), improve endothelial-dependent vasodilation, and decrease markers of systemic inflammation, such as C-reactive protein (CRP) and interleukin-6 (IL-6) [58]. Additionally, vitamin C enhances nitric oxide (NO) bioavailability and supports endothelial nitric oxide synthase (eNOS) activity, contributing to improved vascular function—a critical factor in preventing diabetic complications, such as nephropathy and retinopathy.
- Vitamin E: Vitamin E is a lipophilic antioxidant composed of eight isoforms (α-, β-, γ-, and δ-tocopherols and tocotrienols), with α-tocopherol being the most biologically active and extensively studied. It protects membrane lipids from peroxidation, interrupts lipid radical chain reactions, and modulates cellular signaling cascades. In T2DM, vitamin E has demonstrated the capacity to modulate glucose homeostasis by enhancing glucose transporter type 4 (GLUT4) translocation to the cell membrane and preserving insulin receptor substrate-1 (IRS-1) activity. Furthermore, it inhibits NF-κB activation, thereby reducing the expression of pro-inflammatory cytokines, such as TNF-α and IL-1β. Clinical trials have reported modest improvements in glycemic control and lipid profiles following high-dose α-tocopherol supplementation, although interindividual variability and baseline oxidative stress levels significantly influence the outcomes [59,60].

### 3.2. Bioactivity of Plant-Derived Antioxidants

The bioactivity of plant-derived antioxidants is determined by their ability to interact with multiple cellular and molecular targets, influencing critical physiological processes, such as oxidative stress regulation, inflammatory response modulation, and insulin signaling optimization. These bioactive compounds exert pleiotropic effects through direct radical scavenging, enhancement of endogenous antioxidant defenses, and modulation of signaling pathways implicated in metabolic homeostasis [42]. Given their multifaceted roles, plant-derived antioxidants are increasingly recognized as potential therapeutic agents for the prevention and management of chronic metabolic disorders, particularly type 2 diabetes mellitus (T2DM) and its associated complications [17].

#### 3.2.1. Modulation of Oxidative Stress

Oxidative stress, defined as a pathological imbalance between reactive oxygen species (ROS) production and antioxidant defense mechanisms, is a pivotal contributor to the onset and progression of T2DM. Persistent oxidative stress not only exacerbates pancreatic β-cell dysfunction due to their inherently low antioxidant capacity but also promotes insulin resistance through oxidative damage to insulin-sensitive tissues [20]. Plant-derived antioxidants mitigate oxidative stress via two primary mechanisms: direct ROS neutralization and upregulation of endogenous antioxidant enzyme systems.

##### Direct Scavenging of ROS

Several plant-derived antioxidants exhibit robust free radical scavenging properties owing to their conjugated ring structures and hydroxyl functional groups, which facilitate hydrogen atom donation and electron transfer to neutralize ROS [4,44].

- Flavonoids: Quercetin and catechins interact with superoxide anions (O_2_^−^), hydroxyl radicals (OH•), and hydrogen peroxide (H_2_O_2_), effectively reducing oxidative damage to lipids, proteins, and DNA.
- Carotenoids: β-carotene and lycopene exert singlet oxygen (^1^O_2_) quenching activity, thereby protecting polyunsaturated fatty acids from peroxidation, a key process in diabetic complications, such as nephropathy and neuropathy [39].

##### Enhancement of Endogenous Antioxidant Enzymes

In addition to direct ROS neutralization, plant-derived antioxidants enhance cellular defense systems by upregulating key endogenous antioxidant enzymes, including superoxide dismutase (SOD), catalase (CAT), and glutathione peroxidase (GPx).

- Resveratrol: This polyphenol from grapes and red wine activates nuclear factor erythroid 2-related factor 2 (Nrf2), a master regulator of antioxidant defense, leading to increased expression of SOD and CAT, thereby reducing oxidative damage in pancreatic β-cells and insulin-sensitive tissues [53].
- Curcumin: The principal bioactive compound in turmeric enhances GPx activity and prevents lipid peroxidation by modulating the Nrf2/Keap1 pathway, contributing to improved glucose homeostasis and β-cell protection [61].

By mitigating oxidative stress, plant-derived antioxidants safeguard pancreatic β-cell integrity, enhance insulin signaling, and attenuate the development of diabetes-related complications, including retinopathy, nephropathy, and cardiovascular disease.

#### 3.2.2. Anti-Inflammatory Effects

Chronic low-grade inflammation is a hallmark of T2DM and a key driver of insulin resistance. Pro-inflammatory cytokines, such as tumor necrosis factor-alpha (TNF-α) and interleukin-6 (IL-6), interfere with insulin receptor signaling, impairing glucose uptake and exacerbating metabolic dysregulation. Plant-derived antioxidants exert anti-inflammatory effects by modulating key inflammatory pathways, including nuclear factor-kappa B (NF-κB) and mitogen-activated protein kinase (MAPK), thereby reducing systemic inflammation and improving insulin sensitivity [6].

##### Inhibition of the NF-κB Pathway

NF-κB is a central regulator of inflammation controlling the transcription of pro-inflammatory mediators, such as TNF-α, IL-6, and inducible nitric oxide synthase (iNOS). Plant-derived antioxidants inhibit NF-κB activation through various mechanisms.

- Quercetin: Suppresses NF-κB signaling in adipose tissue, reducing TNF-α and IL-6 levels and thereby improving insulin sensitivity in diabetic patients [45].
- Anthocyanins: These pigments from berries and red grapes inhibit NF-κB activation and decrease circulating C-reactive protein (CRP) levels, a key biomarker of systemic inflammation [61].

##### Modulation of the MAPK Pathway

The MAPK pathway, including extracellular signal-regulated kinase (ERK), c-Jun N-terminal kinase (JNK), and p38 MAPK, is crucial for inflammatory responses and insulin resistance.

- Curcumin: Attenuates inflammation by inhibiting JNK and p38 MAPK phosphorylation, reducing cytokine-mediated insulin resistance [61].
- Epigallocatechin gallate (EGCG): A catechin from green tea that downregulates ERK and JNK activation, mitigating inflammation and oxidative stress in metabolic tissues [62].

##### Reduction of Inflammasome Activation

Inflammasomes, particularly the NLRP3 inflammasome, are critical regulators of IL-1β and IL-18 production, mediators of metabolic inflammation.

- Resveratrol: Suppresses NLRP3 inflammasome activation by reducing mitochondrial ROS production, thereby attenuating inflammatory damage in insulin-sensitive tissues [53].
- Quercetin: Inhibits inflammasome activation and IL-1β release, protecting against inflammation-induced insulin resistance [45].

By targeting inflammatory pathways, plant-derived antioxidants enhance metabolic health, reduce systemic inflammation, and mitigate the progression of T2DM.

#### 3.2.3. Modulation of Insulin Signaling Pathways

Plant-derived antioxidants not only reduce oxidative stress and inflammation but also directly enhance insulin sensitivity by modulating key intracellular pathways involved in glucose metabolism.

- Quercetin: Enhances glucose uptake in skeletal muscle by activating the IRS/PI3K/Akt pathway and promoting GLUT4 translocation to the plasma membrane, facilitating cellular glucose entry [63].
- Resveratrol: Activates AMP-activated protein kinase (AMPK), a crucial regulator of energy homeostasis, promoting glucose uptake and inhibiting hepatic gluconeogenesis, thus improving glycemic control [64].

#### 3.2.4. Gut Microbiota Interactions

Recent research highlights the gut microbiota as a key mediator of metabolic health, with plant-derived antioxidants modulating microbial composition and function. These compounds promote the proliferation of beneficial bacteria, such as Lactobacillus and Bifidobacterium, while inhibiting pathogenic species.

- Polyphenols: Polyphenols are metabolized into bioactive derivatives, such as urolithins and equol, which exhibit potent anti-inflammatory and antioxidant effects, further enhancing systemic metabolic health [65].
- Anthocyanins: Alter gut microbiota composition, favoring an anti-inflammatory profile, which contributes to improved insulin sensitivity and reduced metabolic endotoxemia [66].

Given the intricate interplay between plant-derived antioxidants and metabolic pathways, these bioactive compounds hold promise as therapeutic agents in preventing and managing T2DM and its associated complications.

## 4. Molecular Targets of Antioxidants in Diabetes

Type 2 diabetes mellitus is characterized by chronic insulin resistance, systemic inflammation, and mitochondrial dysfunction, all of which are tightly linked to elevated levels of ROS and oxidative stress. Plant-derived antioxidants exert therapeutic effects not merely through free radical scavenging but by targeting specific intracellular pathways, modulating gene expression, and influencing insulin signaling mechanisms. Understanding these molecular targets is critical for rationally designing antioxidant-based interventions for diabetes management [67].

A primary molecular target of dietary antioxidants is the insulin signaling cascade, particularly the insulin receptor substrate (IRS)/phosphatidylinositol 3-kinase (PI3K)/protein kinase B (Akt) pathway, which regulates glucose uptake, glycogen synthesis, and metabolic homeostasis. In individuals with type 2 diabetes mellitus, this pathway is often impaired due to chronic inflammation, oxidative stress, and serine phosphorylation of IRS proteins, leading to diminished downstream signaling and glucose intolerance. Plant-derived polyphenols, including resveratrol, quercetin, and epigallocatechin gallate (EGCG), have been extensively studied for their capacity to enhance Akt phosphorylation, preserve IRS-1/2 activity, and stimulate GLUT4 translocation to the plasma membrane, thereby facilitating glucose uptake in skeletal muscle and adipose tissue [68]. One critical mechanism involves the inhibition of protein tyrosine phosphatase 1B (PTP1B), a known negative regulator of insulin receptor activity. By downregulating PTP1B, these antioxidants sustain insulin receptor phosphorylation and prevent desensitization of insulin signaling [69].

In addition, in vitro studies and diabetic animal models have demonstrated that resveratrol-rich interventions significantly lower fasting glucose and enhance insulin sensitivity via this mechanism. In parallel, several flavonoids activate AMP-activated protein kinase (AMPK), a main regulator of cellular energy homeostasis. Activation of AMPK not only promotes fatty acid oxidation and mitochondrial biogenesis but also inhibits hepatic gluconeogenesis, thereby improving glycemic control. Also, compounds like baicalein, fisetin, and luteolin have been reported to upregulate AMPK phosphorylation, reduce hepatic glucose output, and restore insulin responsiveness in both hepatic and peripheral tissues. These dual effects on both insulin-dependent and insulin-independent glucose regulation pathways underscore the multifaceted therapeutic potential of plant antioxidants in managing metabolic dysfunctions associated with type 2 diabetes mellitus [69].

Oxidative stress plays a central role in the development and progression of T2DM by damaging pancreatic β-cells, impairing insulin signaling, and accelerating systemic inflammation. In diabetic states, the overproduction of reactive oxygen species (ROS) overwhelms endogenous defense mechanisms, leading to lipid peroxidation, protein carbonylation, and DNA damage. Plant-derived antioxidants counteract this imbalance through two primary mechanisms: direct ROS scavenging and the upregulation of endogenous antioxidant enzymes. Key polyphenolic compounds, such as curcumin, kaempferol, and rutin, have demonstrated the ability to activate the nuclear factor erythroid 2–related factor 2 (Nrf2) pathway, which controls the transcription of antioxidant response elements (AREs). Upon activation, Nrf2 dissociates from its cytoplasmic repressor Keap1, translocates to the nucleus, and binds to AREs to stimulate the expression of key detoxifying and antioxidant enzymes, including superoxide dismutase (SOD), catalase (CAT), and glutathione peroxidase (GPx). These enzymes play critical roles in neutralizing superoxide radicals, decomposing hydrogen peroxide, and maintaining glutathione homeostasis, thereby reducing oxidative load in insulin-sensitive tissues. Importantly, this antioxidant defense extends to the preservation of pancreatic β-cell viability, a critical target in T2DM management. β-cells are particularly vulnerable to oxidative stress due to their low expression of intrinsic antioxidant enzymes. Antioxidants, such as anthocyanins derived from blueberries, blackcurrants, and chokeberries, have shown in rodent models the ability to reduce lipid peroxidation, suppress nitric oxide overproduction, and restore SOD and GPx activity in pancreatic and hepatic tissues [69,70]. These effects not only mitigate β-cell apoptosis but also sustain insulin secretion capacity, contributing to better glycemic control. Together, the modulation of oxidative stress by plant antioxidants underscores their therapeutic value not merely as scavengers of free radicals but as metabolic regulators capable of reprogramming redox-sensitive gene networks in diabetes.

Chronic low-grade inflammation is a critical contributor to the pathogenesis of type 2 diabetes mellitus, acting as a key mediator of insulin resistance and β-cell dysfunction. A central axis in this inflammatory response is the nuclear factor kappa B (NF-κB) signaling pathway, which regulates the transcription of pro-inflammatory cytokines, such as tumor necrosis factor-alpha (TNF-α), interleukin-6 (IL-6), and interleukin-1β (IL-1β). These cytokines impair insulin signaling by promoting serine phosphorylation of insulin receptor substrates and interfering with glucose transporter expression [70]. Plant-derived antioxidants exert potent anti-inflammatory effects by targeting upstream regulators of NF-κB, particularly the IκB kinase β (IKKβ) complex, which initiates NF-κB activation via phosphorylation of the inhibitory IκB protein. Polyphenols, such as luteolin, apigenin, and resveratrol, have been shown to inhibit this process, preventing the nuclear translocation of NF-κB p65 subunits and thereby downregulating the transcription of inflammatory genes. This action results in reduced levels of circulating cytokines, diminished oxidative stress, and improved insulin sensitivity. In parallel, these antioxidants also modulate stress-responsive mitogen-activated protein kinases (MAPKs), such as c-Jun N-terminal kinase (JNK) and p38 MAPK, which are activated in response to metabolic stress and play pivotal roles in inflammation-induced insulin resistance. Activation of JNK leads to serine phosphorylation of IRS-1, an inhibitory modification that disrupts insulin signal transduction. Flavonoids, such as naringenin and kaempferol, have demonstrated the ability to suppress JNK and p38 phosphorylation, preserving IRS-1 tyrosine phosphorylation and insulin receptor functionality [71]. Together, these mechanisms illustrate how plant-derived antioxidants not only suppress inflammatory mediators at the transcriptional level but also interrupt intracellular signaling cascades that lead to metabolic dysregulation. Their pleiotropic action supports the use of phytochemicals as anti-inflammatory insulin sensitizers in the dietary management of type 2 diabetes mellitus.

Mitochondrial dysfunction is increasingly recognized as a central feature of insulin resistance and type 2 diabetes mellitus, particularly in skeletal muscle, liver, and pancreatic β-cells. Diabetic tissues often exhibit impaired oxidative phosphorylation, decreased mitochondrial DNA content, and altered dynamics, resulting in reduced ATP production and increased production of ROS. Recent evidence indicates that plant-derived antioxidants can ameliorate these dysfunctions by promoting mitochondrial biogenesis and improving mitochondrial efficiency. Polyphenols, such as resveratrol and berberine, have been shown to activate the PGC-1α/SIRT1/TFAM signaling axis, a critical pathway for mitochondrial biosynthesis and respiratory function. Peroxisome proliferator-activated receptor gamma coactivator 1-alpha (PGC-1α) regulates mitochondrial gene expression, while SIRT1, a NAD^+^-dependent deacetylase, promotes mitochondrial transcription and stress resilience. Mitochondrial transcription factor A (TFAM) is required for mitochondrial DNA replication and transcription. Upregulation of these genes results in enhanced mitochondrial density, improved fatty acid oxidation, and more efficient glucose utilization, collectively contributing to reductions in hyperglycemia, insulin resistance, and dyslipidemia [72]. In addition to mitochondrial biogenesis, many plant antioxidants also modulate autophagy, a catabolic process critical for cellular homeostasis and the removal of dysfunctional organelles and misfolded proteins. Compounds, such as quercetin, epicatechins, and kaempferol, have been reported to induce autophagy via the AMPK-mTOR and SIRT1-FOXO pathways, thereby promoting the clearance of lipid droplets and reducing endoplasmic reticulum stress, a known contributor to insulin resistance and β-cell apoptosis [73]. By restoring mitochondrial integrity and enhancing autophagic flux, plant antioxidants support not only cellular energy balance but also insulin signaling fidelity. These effects underscore their broader role as metabolic modulators with the potential to counteract the bioenergetic deficits seen in type 2 diabetes mellitus.

Adipokine hormones secreted by adipose tissue play crucial roles in regulating glucose metabolism, lipid homeostasis, and systemic insulin sensitivity. Among these, adiponectin is of particular interest due to its insulin-sensitizing, anti-inflammatory, and cardioprotective properties. In type 2 diabetes mellitus, circulating adiponectin levels are typically reduced, contributing to insulin resistance and chronic inflammation. Plant-derived antioxidants, including kaempferol, genistein, and anthocyanin-rich extracts, have been shown to upregulate adiponectin expression and restore its receptor activity, thereby enhancing insulin responsiveness in adipose and skeletal muscle tissues [74]. Simultaneously, these compounds improve leptin sensitivity, which is often disrupted in obesity-linked type 2 diabetes mellitus, leading to appetite dysregulation and hyperphagia. Restoring leptin signaling in the hypothalamus via antioxidant-mediated suppression of inflammation and oxidative stress contributes to improved energy balance and glycemic control. Isoflavones, such as genistein, have been shown to modulate hypothalamic signaling pathways, reducing resistance to leptin and suppressing neuroinflammatory cascades. Beyond adipose tissue, plant antioxidants significantly influence the gut microbiota–brain–liver axis, a regulatory network critical to metabolic homeostasis. Polyphenols, such as quercetin, catechins, and chlorogenic acid, have been shown to enhance the growth of beneficial microbial taxa, including *Akkermansia muciniphila*, *Bifidobacteria*, and *Faecalibacterium prausnitzii*. These shifts in microbial composition are associated with increased production of short-chain fatty acids (SCFAs), particularly butyrate and propionate, which improve intestinal barrier integrity, reduce metabolic endotoxemia, and lower systemic inflammation, all factors implicated in the pathogenesis of insulin resistance [74,75]. Collectively, these effects reflect a multi-organ modulatory role of plant antioxidants in type 2 diabetes mellitus, wherein adipokine regulation and microbiome remodeling act in concert to restore metabolic flexibility, reduce low-grade inflammation, and support glucose homeostasis.

In summary, plant-derived antioxidants target multiple molecular mechanisms central to the pathogenesis of type 2 diabetes mellitus, including impaired insulin signaling, oxidative stress, inflammation, mitochondrial dysfunction, and dysregulated adipokine signaling. Through the modulation of pathways like IRS/PI3K/Akt, Nrf2-Keap1, NF-κB, and AMPK, as well as influencing mitochondrial biogenesis, autophagy, and the gut microbiota, these phytochemicals offer a multi-pronged approach to restoring metabolic homeostasis. Their pleiotropic actions not only alleviate hyperglycemia and insulin resistance but also provide cytoprotective and anti-inflammatory effects, underscoring their therapeutic potential as adjuncts in the dietary management and prevention of type 2 diabetes mellitus.

To facilitate a comparative understanding of the main antioxidant classes discussed, Table 1 provides a synthesis of their key molecular targets, mechanisms of action, clinical evidence, limitations, and potential applications in the context of T2DM

## 5. Resveratrol: Mechanisms and Clinical Evidence

### 5.1. Antioxidant Properties (Nrf2/Keap1 Pathway)

Oxidative stress plays a critical role in T2DM pathogenesis by damaging cells and exacerbating insulin resistance. Resveratrol exhibits potent antioxidant effects in diabetic models, largely through activation of the Nrf2/Keap1 pathway [76,77]. Nrf2 is a transcription factor that upregulates cellular antioxidant defenses; resveratrol has been shown to increase Nrf2 protein levels and downstream antioxidant gene expression in high-glucose and high-fructose diet models. By stabilizing Nrf2 (for example, via interference with its inhibitor Keap1), resveratrol enhances the expression of cytoprotective enzymes, such as heme oxygenase-1 (HO-1) and glutathione S-transferase (GST), thereby mitigating ROS accumulation. This Nrf2-mediated antioxidant action is thought to underlie many metabolic benefits of resveratrol in diabetes [78].

In addition, resveratrol can directly scavenge free radicals due to its polyphenolic structure, further reducing oxidative damage in pancreatic β-cells and peripheral tissue. Through these mechanisms, resveratrol helps break the cycle of chronic hyperglycemia-induced oxidative stress in T2DM [78].

### 5.2. Anti-Inflammatory Effects (NF-κB and Pro-Inflammatory Cytokines)

Chronic low-grade inflammation is a hallmark of T2DM, and resveratrol exerts anti-inflammatory effects by targeting key inflammatory pathways. Notably, resveratrol inhibits the activation of nuclear factor kappa B (NF-κB), a transcription factor that controls the expression of many pro-inflammatory genes [79]. In diabetic animal models, resveratrol administration significantly decreased NF-κB activity, leading to lower levels of tumor necrosis factor-α (TNF-α), interleukin-6 (IL-6), IL-1β, and other cytokines and chemokines [80].

These changes translate into reduced tissue inflammation; for example, resveratrol attenuated inflammatory protein expression (TNF-α, IL-6, COX-2) in diabetic neuropathy models, improving neuroinflammation and functional deficits [5]. Resveratrol’s anti-inflammatory action is partly mediated by SIRT1, a deacetylase activated by resveratrol that can bind and suppress NF-κB’s p65 subunit, leading to the inhibition of its transcriptional activity [78].

By blocking NF-κB-driven gene transcription, resveratrol downregulates adhesion molecules (ICAM-1, VCAM-1) and inflammatory enzymes, curbing the recruitment of macrophages and inflammatory cells to tissues [81]. As a result, pancreatic islets, adipose tissue, and the vasculature experience less inflammatory stress. This anti-inflammatory property of resveratrol has been confirmed across multiple diabetic models, where it consistently reduces the expression of TNF-α, IL-1β, IL-6, and other NF-κB downstream mediators. By dampening inflammation, resveratrol helps improve insulin signaling and prevents damage to insulin-producing cells in T2DM [82].

### 5.3. Effects on Insulin Sensitivity (AMPK/SIRT1 and IRS/PI3K/Akt Signaling)

A key anti-diabetic mechanism of resveratrol is the improvement of insulin sensitivity in insulin-resistant tissues. Resveratrol activates cellular energy sensor pathways, such as AMP-activated protein kinase (AMPK) and SIRT1, which in turn enhance insulin signaling. In diabetic mice, resveratrol treatment significantly improved insulin sensitivity as measured by HOMA-IR, an effect linked to AMPK activation [81]. AMPK activation promotes glucose uptake (e.g., by increasing GLUT4 translocation) and fatty acid oxidation, counteracting insulin resistance.

Concurrently, resveratrol-induced activation of SIRT1 (a NAD^+^-dependent deacetylase) appears to augment insulin’s action in liver and adipose tissue SIRT1 activation as resveratrol improves insulin signaling through multiple routes; it deacetylates and activates key metabolic regulators (FOXO1, PGC-1α), and it can suppress negative regulators of insulin signaling [83]. For instance, resveratrol may inhibit protein tyrosine phosphatase 1B (PTP1B), a phosphatase that attenuates insulin receptor signaling, thereby enhancing IRS-1/PI3K/Akt pathway activity [78].

In insulin-resistant animal models, resveratrol restored the NAD^+^/NADH ratio and SIRT1 levels, leading to improved insulin sensitivity. Human cell studies likewise show that resveratrol’s activation of AMPK/SIRT1 can upregulate the insulin signaling cascade (IRS → PI3K → Akt), improving glucose uptake in muscle and adipose cells [78,84].

Collectively, these molecular actions translate into lower blood glucose and insulin levels in vivo. In fact, a comprehensive analysis by Liu et al. found that resveratrol significantly reduced blood glucose and improved insulin sensitivity in diabetic patients without major adverse effects [82]. Thus, resveratrol acts as an insulin sensitizer by modulating AMPK/SIRT1 and downstream insulin signaling pathways that are often impaired in T2DM.

### 5.4. Mitochondrial Function and Energy Metabolism (PGC-1α Activation)

Resveratrol favorably impacts mitochondrial function and energy metabolism, which is crucial, as mitochondrial dysfunction contributes to insulin resistance in T2DM. Through its activation of SIRT1 and AMPK, resveratrol upregulates PGC-1α (Peroxisome Proliferator-Activated Receptor Gamma Coactivator-1α), a master regulator of mitochondrial biogenesis and oxidative.

Studies in obese and diabetic animals demonstrate that resveratrol increases PGC-1α activity, leading to enhanced mitochondrial biogenesis and respiratory capacity in skeletal muscle and the liver [81]. This helps cells oxidize glucose and fatty acids more efficiently. Improved mitochondrial function reduces ectopic lipid accumulation and improves insulin action. For example, in high-fat-diet models, resveratrol treatment increases mitochondrial gene expression and enzyme activities, which is associated with improved whole-body glucose metabolism [83].

Additionally, resveratrol’s activation of SIRT1–PGC-1α signaling can induce the expression of antioxidant enzymes (via FOXO and PPAR pathways), protecting mitochondria from hyperglycemia-induced oxidative damage [78]. By preserving mitochondrial integrity and function, resveratrol supports better energy utilization and prevents the energy imbalances seen in diabetic tissues.

Notably, resveratrol has been likened to a caloric restriction mimetic, as it triggers molecular responses (AMPK/SIRT1 activation, mitochondrial biogenesis) similar to those observed under caloric restriction, which is known to improve metabolic health. In muscle cells, resveratrol also promotes the Akt pathway and GLUT4 translocation, thereby enhancing glucose uptake and utilization [83,84].

Overall, the effect of resveratrol on mitochondria is two-fold; it increases the number and efficiency of mitochondria(via PGC-1α) and decreases mitochondrial oxidative stress, thus tackling a root cause of insulin resistance in T2DM [84].

### 5.5. β-Cell Protection and Insulin Secretion

Preservation of pancreatic β-cell function is vital for T2DM management, and resveratrol has shown protective effects on β-cells through anti-apoptotic and insulinotropic actions [85]. Chronic hyperglycemia and inflammation in T2DM can induce β-cell apoptosis and dysfunction. Resveratrol combats this by inhibiting pathways that lead to β-cell death. For instance, by suppressing NF-κB and the inflammatory cascade, resveratrol downregulates pro-apoptotic signals in pancreatic islets, thereby reducing β-cell stress and improving cell survival [86]. This NF-κB inhibition is linked to the reduced expression of β-cell apoptotic markers, thereby improving β-cell survival. In streptozotocin-induced diabetic models (a widely used model of β-cell damage), resveratrol treatment preserved β-cell mass and function, partly by blocking oxidative and inflammatory damage to the pancreas [87].

Resveratrol also influences insulin secretion dynamics. It has been found to potentiate glucose-stimulated insulin secretion (GSIS) under certain conditions. Mechanistic studies revealed that resveratrol acutely inhibits β-cell ATP-sensitive K^+^ channels (K_ATP) and voltage-dependent K^+^ channels (K_V) on the β-cell membrane, which leads to prolonged cell depolarization and enhanced insulin exocytosis [87,88].

In pancreatic islet experiments, low-dose resveratrol increased insulin release through this K^+^ channel blockade, an effect similar to that of some sulfonylurea drugs. Resveratrol’s activation of SIRT1 in β-cells further contributes to insulin secretory capacity, as SIRT1 suppresses the expression of uncoupling protein 2 (UCP2), a mitochondrial protein that normally reduces ATP production in β-cells, thereby enhancing insulin secretion [88]. By repressing UCP2, resveratrol via SIRT1 increases ATP availability, facilitating glucose-stimulated insulin secretion. These combined actions result in more effective insulin release in response to glucose, thereby improving glycemic control. It is worth noting that the effect of resveratrol on insulin secretion can be context-dependent (very high concentrations in vitro have been reported to inhibit secretion, potentially to prevent β-cell exhaustion) [89]. However, in diabetic settings in vivo, resveratrol’s net effect tends to be protective; it prevents β-cell apoptosis while normalizing insulin secretion dynamics. Overall, resveratrol helps maintain functional β-cell mass as it shields β-cells from oxidative and inflammatory injury and supports their insulin output, which is crucial for slowing T2DM progression [86,88].

### 5.6. Clinical Evidence from Human Studies

#### 5.6.1. Findings from Meta-Analyses and Clinical Trials

The potential benefits of resveratrol in T2DM have been evaluated in numerous clinical trials, and several systematic reviews/meta-analyses summarize these outcomes. Current evidence from human studies suggests that resveratrol supplementation can modestly improve glycemic control and some cardiometabolic parameters in type 2 diabetic patients, although results vary between trials [90]. A 2022 meta-analysis of 17 randomized controlled trials (RCTs) involving 871 patients concluded that resveratrol supplementation significantly improved glycemic control in T2DM compared to placebo, particularly through reductions in fasting blood glucose (FBG) and HbA1c, with greater effects at higher doses and longer durations [90]. Similarly, another comprehensive meta-analysis in 2022 including 19 RCTs (1151 patients) found that high-dose resveratrol supplementation significantly lowered blood glucose and blood pressure, reinforcing its potential cardiometabolic benefits [91]. Importantly, these benefits were achieved with a good safety profile, as no serious adverse effects were reported across studies [89]. However, not all clinical trials have demonstrated positive results, and some outcomes, such as lipid profiles, have shown inconsistencies [92]. Meta-analytic data highlight significant heterogeneity among studies, which may stem from differences in resveratrol doses, treatment durations, and patient populations [89]. The following sections explore the clinical evidence for specific glycemic, metabolic, and cardiovascular outcomes and the conditions under which resveratrol appears most effective.

#### 5.6.2. Effects on Glycemic Control (HbA1c, Glucose, Insulin Sensitivity)

Resveratrol supplementation has demonstrated notable improvements in several indices of glycemic control in T2DM patients. Among these, fasting blood glucose (FBG) is the most consistently improved parameter.

#### 5.6.3. Fasting Blood Glucose (FBG)

Several meta-analyses report that resveratrol-treated groups exhibit significantly lower FBG levels compared to placebo-treated controls [93]. The magnitude of FBG reduction appears to be dose-dependent:

- High-dose regimens (≥500–1000 mg/day) reduced FBG by approximately 0.7–1.0 mmol/L (~13–18 mg/dL) [79].
- Lower doses (<500 mg/day) yielded smaller or negligible reductions.
- In one meta-analysis, trials using ≥1000 mg/day achieved a mean FBG drop of ~18.8 mg/dL [93].

These improvements in fasting glucose likely reflect enhanced insulin-mediated glucose uptake and/or reduced hepatic glucose production [91].

#### 5.6.4. Glycated Hemoglobin (HbA1c)

HbA1c, a marker of long-term glycemic control, has shown modest improvements with resveratrol supplementation. While early meta-analyses with shorter-duration trials often reported no significant effect, more recent studies suggest otherwise.

- A 2022 meta-analysis detected a small but significant improvement in HbA1c (~0.4% absolute reduction at 3 months) with resveratrol vs. placebo [90].
- One RCT found that 3 months of resveratrol (250 mg/day), when added to standard anti-diabetic therapy, led to a statistically significant decrease in HbA1c [94].
- Another trial using 1 g/day for 45 days reported a reduction in both HbA1c and fasting glucose [79].

These findings suggest that while resveratrol is not a substitute for conventional glucose-lowering drugs, it may provide incremental benefits in long-term glycemic control, particularly when used for extended durations at adequate doses [93].

#### 5.6.5. Insulin Sensitivity and HOMA-IR

Resveratrol has also been shown to enhance insulin sensitivity, aligning with preclinical findings. Several trials report reductions in fasting insulin levels and HOMA-IR (Homeostasis Model Assessment of Insulin Resistance) in resveratrol-treated patients.

- A meta-analysis of five trials (153 patients) found that resveratrol significantly lowered HOMA-IR, indicating improved insulin action (pooled decrease in HOMA-IR by ~0.5 units).
- Concurrently, fasting insulin concentrations decreased in response to resveratrol supplementation [92,95].
- In a placebo-controlled trial, resveratrol (1 g/day for 6 weeks) led to a ~20% reduction in fasting insulin and insulin resistance index [79].
- Another small-scale study (5 mg twice daily) observed improved insulin sensitivity and increased Akt phosphorylation in platelets, a surrogate for insulin signaling activity [89].

These findings suggest that resveratrol provides an additive metabolic benefit, even in patients already receiving standard anti-diabetic medications [93]. Mechanistically, these improvements in insulin sensitivity are attributed to resveratrol’s activation of AMPK, suppression of inflammation, and enhanced mitochondrial function, all of which counteract insulin resistance [79].

However, in patients with relatively well-controlled or early-stage T2DM, some studies have not detected a significant resveratrol effect on insulin sensitivity, potentially due to ceiling effects or suboptimal dosing. Overall, human clinical data indicate that resveratrol modestly improves glycemic parameters, including lowering fasting and postprandial glucose and enhancing insulin responsiveness, particularly when administered at higher doses for longer durations [91].

### 5.7. Impact on Lipid Profile and Cardiovascular Parameters

The effect of resveratrol on lipid profiles in T2DM patients is less pronounced than its glucose-lowering effect, with mixed findings in the literature. Some clinical trials and meta-analyses have reported modest improvements in certain lipid parameters, while others found no significant changes [90].

For instance, a 2022 meta-analysis by Abdelhaleem et al. reported that resveratrol significantly reduced total cholesterol (mean decrease ~5–6 mg/dL) in diabetic patients [90]. However, changes in LDL-C and triglycerides were not statistically significant in most pooled analyses [96]. Similarly, a meta-analysis by Wei Gu et al. found no improvement in triglyceride or HDL-C levels, even at high doses of resveratrol [79,89].

One RCT observed a small increase in HDL cholesterol (+2 mg/dL) after 45 days of high-dose resveratrol (1 g/day) [79], but this finding has not been consistently replicated across other studies. On balance, resveratrol’s direct effects on lipid metabolism in humans appear modest; it may slightly reduce total and/or LDL cholesterol in some cases, but overall lipid profile changes (including triglycerides and HDL) remain variable and often not significant. These inconsistencies may arise due to differences in baseline lipid levels, concurrent medications, or the relatively short duration of many trials (which are often insufficient to observe significant cholesterol changes, as these typically require longer-term interventions) [93].

Beyond blood lipids, resveratrol has shown more pronounced benefits on blood pressure and vascular function, which are particularly relevant for T2DM patients, given their high prevalence of hypertension. Several clinical studies have documented reductions in blood pressure with resveratrol supplementation.

- Meta-analyses indicate that resveratrol (particularly at higher doses) was associated with significantly lower systolic and diastolic blood pressure compared to placebo [90].
- Pooled data indicate an average systolic BP reduction of 5–8 mmHg and a diastolic reduction of ~2–4 mmHg in resveratrol-treated diabetics [96].
- One meta-analysis found a mean systolic BP drop of 7.97 mmHg and a diastolic drop of 3.55 mmHg with resveratrol vs. the control [79].

These are clinically relevant improvements in blood pressure, likely reflecting resveratrol’s vasodilatory and endothelial-protective properties, which are mediated through increased nitric oxide bioavailability and reduced arterial inflammation.

Improved blood pressure control may contribute to resveratrol’s reported benefits with regard to diabetic cardiovascular outcomes in some studies, such as enhanced circulation in diabetic foot ulcers and decreased arterial stiffness.

In contrast to blood pressure, resveratrol’s effect on body weight or adiposity indices appears minimal. RCTs generally show no significant change in body weight or waist circumference due to resveratrol supplementation [96], which suggests its metabolic benefits are not due to weight loss.

Taken together, while resveratrol is not a potent lipid-modulating agent, it may confer cardiovascular benefits in T2DM by modestly improving cholesterol levels (in select cases) and significantly reducing blood pressure. These cardiometabolic effects support the idea of resveratrol as an adjunct therapy to improve overall risk factors in diabetes [93].

### 5.8. Inflammation and Oxidative Stress Biomarkers in Patients

Consistent with its mechanistic actions, resveratrol supplementation in T2DM patients has been shown to ameliorate biomarkers of inflammation and oxidative stress in clinical studies.

A recent meta-analysis (2024) focusing on inflammatory and oxidative outcomes in diabetic patients found that resveratrol significantly lowers circulating inflammatory markers, particularly C-reactive protein (CRP) [94]. CRP, a key marker of systemic inflammation, was reduced with an average effect size (SMD) of about −1.4, indicating a clinically relevant anti-inflammatory effect [94].

Interestingly, while the meta-analysis showed clear reductions in CRP, resveratrol did not significantly reduce IL-6 or TNF-α levels on average [94]. However, there was a trend toward lower IL-6 levels (*p* = 0.06), suggesting that longer interventions may be necessary to detect statistically significant changes in these pro-inflammatory cytokine. The lack of statistical significance for cytokine reductions could also be attributed to high variability between studies or insufficient duration to observe meaningful shifts in inflammatory pathways [97].

Beyond inflammation, resveratrol’s antioxidative effects are reflected in reductions in oxidative stress biomarkers. The 2024 meta-analysis reported significant decreases in lipid peroxidation products, including malondialdehyde (MDA) and 8-isoprostanes, in resveratrol-treated groups. These biomarkers reflect oxidative damage to lipids, a process exacerbated by hyperglycemia and chronic inflammation in poorly controlled diabetes [96].

Additionally, resveratrol supplementation led to improvements in endogenous antioxidant defenses, particularly through increases in glutathione peroxidase (GPx) and catalase levels in T2DM patients. These findings suggest that resveratrol enhances the body’s intrinsic antioxidant capacity, likely through Nrf2 pathway activation, as previously discussed [90].

Some studies also assessed superoxide dismutase (SOD) activity and total antioxidant capacity, although the meta-analysis did not find significant changes in these markers, likely due to high heterogeneity across clinical trials [94].

Overall, the human clinical evidence supports that resveratrol can attenuate both pro-inflammatory and pro-oxidant states in T2DM. Reductions in CRP and oxidative stress biomarkers suggest a potential role for resveratrol in mitigating diabetes-related complications, as chronic inflammation and oxidative damage drive atherosclerosis, neuropathy, and other long-term diabetic sequelae. These findings further reinforce the potential of resveratrol as a therapeutic adjuvant, targeting not just glycemic control but also the underlying inflammatory and oxidative milieu of diabetes [97].

### 5.9. Dose–Response Relationships and Safety Considerations

Clinical evidence suggests a dose–response relationship with resveratrol in T2DM, where higher doses and longer durations tend to yield greater metabolic improvements. Human trials have tested doses from 5 mg/day up to 3 g/day, but meta-analyses indicate that a threshold of 300–500 mg/day is required for consistent benefits [96]. Subgroup analyses reveal that doses below 100 mg/day show no significant effects, while doses ≥500 mg/day are associated with better glycemic control, including significant reductions in HbA1c and fasting glucose [91]. The most pronounced benefits appear with doses of ≥1000 mg/day, particularly in terms of blood pressure reduction and insulin sensitivity.

Regarding safety, resveratrol is generally well-tolerated, with no serious adverse effects reported in diabetic patients. The most common side effects include mild gastrointestinal discomfort (e.g., nausea, diarrhea), typically observed at higher doses (>1 g/day) [96]. Studies using 1 g/day for several months found no organ toxicity and good adherence. Even doses up to 5 g/day in non-diabetic populations have been well-tolerated, although gastrointestinal issues may increase at these extremes [95].

Notably, resveratrol does not induce hypoglycemia, as its mechanism enhances insulin sensitivity rather than forcing glucose reduction [91,93]. This makes it a safe adjunct therapy for patients on conventional anti-diabetic medications. While drug interactions are theoretically possible (e.g., via effects on hepatic metabolism enzymes), no clinically relevant interactions have been reported in trials [91,93].

In summary, resveratrol is safe for use in T2DM, and achieving sufficient doses (≥300 mg/day) is key to realizing its metabolic benefits. However, long-term safety beyond one year remains uncertain, requiring further research.

## 6. Curcumin and Its Role in Insulin Sensitivity

Curcumin is a bioactive molecule present in turmeric, a spice extracted from its namesake plant (*Curcuma longa*), that has been widely studied for its medical properties [98]. Curcumin is a polyphenol belonging to the group of curcuminoids, the phenolic compounds that give turmeric its yellow color. This compound has been reported to exhibit antioxidant, antimicrobial, anti-inflammatory, anti-diabetic, hepatoprotective, and antimutagenic properties [99]. The role it presents in improving insulin sensitivity has been particularly reported in the context in T2DM. Evidence suggests that this molecule exerts its effects through several mechanisms, such as anti-inflammatory and antioxidant properties, that can be relevant in managing insulin resistance and oxidative stress associated with T2DM [99,100]. Its particular chemical structure confers curcumin the ability to have many molecular targets. Despite the significant effects it has been proven to have, there are notorious differences between its effectiveness reported in vitro and in vivo. It has been justified that its bioavailability and low water solubility could be reasons behind its lower results in vivo [101].

Curcumin’s ameliorative capacity to enhance insulin sensitivity in T2DM seems to be specially driven by its ability to reduce inflammation and oxidative stress. Noticeably, inflammation is one of the pathogenic factors important for the increased insulin resistance and rise in blood glucose levels that appear in T2DM [102]. Several studies have shown that curcumin can protect against diabetes by decreasing inflammation, as summarized by Gu et al. [99]. Inflammatory mediators, such as IL-6 and TNF-α, were decreased in blood or different cell types in diabetic rats treated with curcumin via suppression of the NF-κB pathway [100]. In addition, curcumin can inhibit JNK phosphorylation and prevent inflammation in diabetic cardiomyopathy [76]. Oxidative stress has also been shown to be related to the pathogenesis of T2DM [103], which makes the antioxidant properties of curcumin another probable mechanism through which this molecule exerts its anti-diabetic effects [99]. A meta-analysis performed by Qin et al. indicated that curcumin had antioxidant effects by reducing the levels of malondialdehyde (MDA), a product of lipid peroxidation, and enhancing superoxide dismutase (SOD) activity [104]. Shafabakhsh et al. additionally showed that curcumin’s oral administration could ameliorate antioxidant indicators in patients with T2DM.

Despite said anti-inflammatory and antioxidant effects, several other mechanisms and signaling pathways seem to be involved in the way curcumin exerts its benefits. First, curcumin metabolites were reported to improve insulin sensitivity by activating the PI3K-AKT-GSK3B and AMPK signaling pathways and suppressing the phosphorylation of ERK/JNK, which are crucial in counteracting insulin resistance, in high-glucose-induced insulin-resistant HepG2 cells [61]. Curcumin has also been shown to increase circulating levels of irisin [105] and adiponectin [106], a myokine and an adipokine, respectively, which can also improve insulin sensitivity. Moreover, curcumin has the potential to upregulate or activate the key regulator PPARγ to extend its capacity to combat insulin resistance. Lee et al. (2022) also reported that curcumin can restore insulin homeostasis in diet-induced obese aged mice by enhancing hepatic insulin-degrading enzyme (IDE) expression and preserving islet integrity [107]. Finally, not only does curcumin directly affect hyperglycemia in the previously stated ways, but it also can reduce other diabetic complications by regulating lipid metabolism [108].

Several clinical trials have supported that curcumin supplementation can improve markers of insulin sensitivity. In one of them, the results suggested that it is an effective antihyperglycemic agent, as shown by decreased blood glucose levels and reduced circulating glycogen synthase kinase-3 beta (GSK-3β) following dietary supplementation with curcumin [109]. Furthermore, Mahdavi et al. systematically reviewed the effects of curcumin supplementation in glycemic control and found a reduction in fasting blood glucose (FBG) and hemoglobin A1c (HbA1c) levels in most studies [110]. A different study in women with polycystic ovary syndrome additionally reported significant reductions in fasting plasma glucose after supplementation with this compound. In conclusion, both animal and clinical studies have presented solid evidence in favor of curcumin’s ability to prevent T2DM. Future directions seem to be focused on improving its bioavailability to tackle the limitations it can present in clinical settings.

## 7. Flavonoids and Metabolic Regulation: A Focus on Quercetin

Flavonoids constitute a diverse subclass of polyphenols with wide-ranging bioactivity in metabolic disorders, including T2DM. Among them, quercetin has been the most extensively studied. However, other flavonoids, such as kaempferol, epicatechin, and naringenin, also exert protective effects via modulation of oxidative stress, inflammation, and insulin signaling. This section highlights the mechanistic role of flavonoids in T2DM, with a particular focus on quercetin as a representative compound. Quercetin, a flavonoid found in onions, apples, berries, broccoli, and tea, has emerged as a compound of significant interest in the complementary approach to type 2 diabetes [111]. This interest stems from its ability to exert multiple actions on the mechanisms underlying insulin resistance and impaired glucose metabolism. The molecular structure of quercetin, featuring a flavonoid core with several hydroxyl groups, underlies the notable antioxidant, anti-inflammatory, and regulatory properties of cell signaling pathways related to glycemic homeostasis [112].

A significant area of research in the field of quercetin’s biological activities is its potential to mitigate oxidative stress, a critical component in the progression of DM2 [113]. Oxidative stress arises from the damage inflicted by ROS on pancreatic β-cells and peripheral tissues. Different preclinical trials in murine models of induced diabetes have demonstrated that quercetin administration enhances the activity of endogenous antioxidant enzymes, including superoxide dismutase, catalase, and glutathione peroxidase [114,115]. This augmentation in antioxidant enzyme activity contributes to a reduction in lipid peroxidation and the subsequent limitation of the cascade of events that leads to chronic inflammation. This antioxidant activity is not only observed in vivo, as in vitro studies have shown how quercetin acts directly as a free radical scavenger, reducing the generation of ROS associated with hyperglycemia [116].

The anti-inflammatory activity of quercetin constitutes another one of its fundamental mechanisms in metabolic regulation [117]. In scenarios of insulin resistance, low-grade inflammation plays a prominent role in the dysfunction of insulin signaling, mainly through cytokines like tumor necrosis factor-alpha (TNF-α) and interleukin-6 (IL-6) [117,118]. Quercetin has been shown to modulate critical signaling pathways involved in the expression of these pro-inflammatory mediators, including the nuclear factor kappa B (NF-κB) and mitogen-activated protein kinase (MAPK) pathways [119]. In several studies utilizing experimental models, quercetin supplementation has been observed to reduce plasma levels of TNF-α and IL-6, with a concomitant positive impact on insulin sensitivity and the integrity of the affected tissues [120,121].

In addition to its antioxidant and anti-inflammatory properties, quercetin has been shown to directly influence insulin signaling and glucose homeostasis through several cellular mechanisms. It has been documented to promote the phosphorylation of the insulin receptor substrate (IRS-1) at tyrosine residues and reduce phosphorylation at serine residues, thereby enhancing the efficiency of the intracellular signaling cascade [122]. This modification in signaling contributes to the increased translocation of glucose transporter type 4 (GLUT4) to the cell membrane, particularly in skeletal muscle, favoring glucose uptake and glycemic control [123]. In contrast, several studies have reported that quercetin activates AMP-activated protein kinase (AMPK), a major regulator of energy metabolism. AMPK activation promotes fatty acid oxidation, attenuates hepatic lipogenesis, and, ultimately, contributes to improved insulin sensitivity and the prevention of associated metabolic dysfunctions [124].

While the preponderance of evidence originates from in vitro and animal studies, human research has also emerged that lends support to the relevance of quercetin as an adjunct in the management of DM2. Preliminary clinical trials have demonstrated that daily supplementation (generally in the range of 500 to 1000 mg) has been associated with modest improvements in insulin resistance and glucose tolerance profiles, as well as reductions in inflammatory markers [124,125]. In addition, studies on patients with metabolic syndrome have shown that after a period of quercetin treatment, there was an improvement in basal glucose levels and total antioxidant capacity [126]. However, the available data remain limited and are sometimes affected by small sample sizes and relatively short intervention durations. Despite these limitations, the consistency of the results in different populations and settings warrants further investigation in larger clinical trials with robust designs to clarify both the actual efficacy of the compound and the optimal doses and potential long-term effects.

One of the challenges associated with the utilization of quercetin in clinical settings pertains to its relatively low bioavailability. Quercetin, a lipophilic flavonoid, exhibits a limited ability to dissolve in aqueous media [127]. Consequently, its intestinal absorption may be compromised, and, following ingestion, the substance undergoes extensive metabolism by intestinal microbiota and hepatic and extrahepatic enzymes [128]. Some studies suggest that glycosylated forms of quercetin or its co-administration with lipids could enhance its absorption [129]. Conversely, others advocate for encapsulations and nanoparticles to improve its stability and prevent premature degradation. The combination of quercetin with other flavonoids, such as catechins or resveratrol, has also been proposed, with the hypothesis that favorable synergism could be achieved in the regulation of glucose metabolism and inflammatory signaling [130].

Another salient aspect pertains to the potential impact of quercetin on the intestinal microbiota. This microbiota is not only implicated in its metabolism; it may also be modulated by the regular consumption of foods rich in phenolic compounds [131]. It has been posited that quercetin may favor the growth of certain beneficial bacteria and attenuate the proliferation of microorganisms that favor systemic inflammation [132]. However, this area of study still requires further empirical support to draw firm conclusions. The prospect of intervening in the microbiota and insulin signaling through dietary guidelines or specific supplements offers novel opportunities for the prevention and management of DM2.

The extant body of data on quercetin suggests its potential to improve metabolic regulation through multiple axes of action. It decreases oxidative stress by boosting endogenous antioxidant systems, moderates inflammatory responses linked to insulin resistance, and promotes insulin signaling through the activation of key cellular pathways, such as AMPK, and the modulation of IRS-1. Although the results of preclinical and early clinical studies are promising, establishing clear dosing guidelines, defining the optimal duration of treatment, and resolving bioavailability issues remain challenges. Notwithstanding these challenges, quercetin exemplifies the potential of plant-derived bioactive compounds to augment the therapeutic armamentarium against type 2 diabetes, whether through dietary integration or the administration of validated supplements in rigorous clinical trials. Beyond quercetin, other flavonoids have also demonstrated promising anti-diabetic effects through distinct molecular mechanisms. Kaempferol, a flavonol found in kale, tea, and broccoli, activates AMPK and promotes PGC-1α expression, contributing to enhanced mitochondrial biogenesis and improved insulin sensitivity. It also exhibits anti-inflammatory properties by downregulating NF-κB signaling and pro-inflammatory cytokines [133]. Epicatechin, abundant in green tea and cocoa, has been shown to enhance endothelial function and stimulate GLUT4 translocation, facilitating glucose uptake in skeletal muscle cells [134]. Additionally, naringenin, a flavanone present in citrus fruits, suppresses JNK phosphorylation and modulates lipid metabolism, thereby improving insulin sensitivity and reducing hepatic steatosis in diabetic models [135]. These findings highlight the shared and complementary pathways through which diverse flavonoids can mitigate metabolic dysfunctions associated with T2DM.

## 8. Anthocyanins and Glycemic Control

Anthocyanins are distinguished not only by their role in the reddish, violet, and blue colors of numerous fruits and vegetables but also by their high therapeutic potential in the context of DM2 [132,136]. Within this family of compounds, various anthocyanidins are recognized (cyanidin, delphinidin, peonidin, malvidin, or pelargonidin, among others), which appear in the form of glycosides linked to sugars, such as glucose or rhamnose. The physicochemical properties and bioavailability of each anthocyanin are determined by the glycosidic bonds and the number and position of hydroxyl groups. These factors also explain the differences observed in the absorption, metabolism, and biological activity profiles between them [137]. Although they are often grouped under the same name, in practice, notable heterogeneity is observed in their efficacy and in the physiological effects they promote.

The antioxidant action of anthocyanins has been extensively documented, and it is among the earliest recognized mechanisms. It has been demonstrated that these molecules are capable of neutralizing free radicals and reducing the formation of reactive oxygen species (ROS), which are the primary causes of lipid peroxidation and oxidative damage to proteins and DNA, even at relatively low concentrations [138]. In the context of DM2, this oxidative stress contributes decisively to pancreatic β-cell dysfunction and the onset of micro- and macrovascular complications, so the ability of anthocyanins to mitigate this process is considered particularly relevant [139]. Research has demonstrated that anthocyanins function as both direct free radical scavengers and modulators of the activity and expression of endogenous enzymes, including superoxide dismutase and catalase, thereby amplifying the body’s antioxidant response [140]. It has been noted that in combination with other flavonoids present in the diet, such as proanthocyanidins, a synergistic effect can be observed that further reinforces cellular protection against oxidative stress [141].

The impact of anthocyanins on inflammation, a process closely linked to DM2, constitutes another fundamental axis of their therapeutic potential. A substantial body of research employing mouse models of obesity and induced diabetes has demonstrated that supplementation with anthocyanins derived from berries (e.g., blueberries, blackberries, strawberries) results in a substantial reduction in the concentration of pro-inflammatory cytokines, including interleukin 6 (IL-6), interleukin 1 beta (IL-1β), and tumor necrosis factor alpha (TNF-α) [142]. This decrease has been shown to positively correlate with an improvement in insulin signaling, suggesting that the attenuation of low-grade inflammation contributes to the partial restoration of insulin sensitivity [143]. Studies in this line indicate that anthocyanins can regulate key inflammatory pathways, such as the nuclear factor kappa B (NF-κB) activation pathway and the mitogen-activated protein kinases (MAPK) pathway [144]. These pathways are essential for the transcription of genes that code for pro-inflammatory mediators. Consequently, the attenuation of these pathways could potentially mitigate overstimulation of the innate immune system and foster a less deleterious metabolic milieu for tissues.

A notable effect of anthocyanins, particularly those derived from select fruits, such as blueberries and red grapes, is their capacity to enhance glycemic homeostasis and insulin sensitivity [145]. This phenomenon occurs through several mechanisms, including the activation of AMP-activated protein kinase. AMPK, regarded as an “energy sensor” within cells, is stimulated, leading to the translocation of glucose transporter type 4 (GLUT4) to the cell membrane in skeletal muscle, thereby augmenting glucose uptake [146]. In addition, the inhibition of enzymes responsible for carbohydrate degradation in the digestive tract, such as α-amylase and α-glucosidase, has been observed. This inhibition reduces the rapid release of glucose into the blood after carbohydrate ingestion and prevents postprandial hyperglycemic peaks [147]. In addition, research involving animal models and cell cultures has indicated that exposure to anthocyanins facilitates the appropriate phosphorylation of the insulin receptor substrate (IRS-1) on tyrosine residues. This, in turn, enhances the transmission of the intracellular insulin signal [148].

In the clinical setting, a multitude of trials have substantiated the efficacy of anthocyanins in enhancing various metabolic parameters in individuals with diabetes or at risk of developing it [149]. Several studies have documented improvements in fasting blood glucose, insulin sensitivity, and lipid profile following supplementation with standardized anthocyanin extracts over the course of several weeks [150]. These data suggest that regular consumption of anthocyanin-rich fruits and vegetables is associated with a reduced risk of developing type 2 diabetes mellitus in the long term. This association could be attributed to the direct effect of anthocyanins on insulin modulation or the displacement of ultra-processed foods in the diet, which are known to be more harmful.

The bioavailability of anthocyanins has been a subject of controversy, as they are typically characterized by limited absorption in the small intestine, with a significant fraction of these compounds reaching the colon [151]. It is well-established that anthocyanin metabolites, such as various phenolic acids resulting from the breakdown of the flavy structure, can retain or even enhance certain biological properties [152]. It has been proposed that regular anthocyanin consumption may exert a prebiotic effect by promoting the proliferation of beneficial bacteria and the production of short-chain fatty acids [153]. These phenomena could positively impact the integrity of the intestinal barrier and, consequently, the systemic inflammation associated with insulin resistance [154]. However, the magnitude of these effects is contingent on variables like the basal composition of the microbiota, the specific dietary source of anthocyanins, and the individual’s health status. Consequently, the necessity of larger controlled trials is evident to specify personalized supplementation guidelines.

A significant challenge to the clinical and nutraceutical application of anthocyanins is their instability. It has been observed that factors like pH, temperature, and the presence of transition metals can affect their structure and accelerate their degradation [155]. Consequently, formulation strategies, such as microencapsulation or the addition of stabilizing compounds (e.g., certain polysaccharides), are being explored with the aim of improving their durability and efficacy in functional products specifically targeted at the control of DM2 [156].

The scientific evidence suggests that anthocyanins could play a valuable role in the management of type 2 diabetes. These compounds have a multifaceted mechanism of action impacting various processes, such as insulin signaling modulation, chronic inflammation reduction, and, potentially, favorable adjustment of the intestinal microbiome. However, the path toward effective and widespread implementation requires further research to more accurately establish doses, the most active chemical forms, the influence of the food matrix, and the ways in which individual factors (genetics, age, dietary habits) modulate the therapeutic response. Notwithstanding, the evidence amassed to date substantiates the promotion of a diet abundant in brightly colored fruits and vegetables, particularly those exhibiting purple hues. This dietary recommendation is predicated on the premise of leveraging the benefits that anthocyanins confer to the general population, with a heightened focus on individuals afflicted with metabolic disorders associated with type 2 diabetes.

## 9. Carotenoids in Diabetes Management

Diabetes mellitus is a multifactorial metabolic disorder characterized by chronic hyperglycemia resulting from defects in insulin secretion, insulin action, or both [157]. The global prevalence of diabetes has reached epidemic proportions, with the International Diabetes Federation reporting over 537 million affected adults in 2021, a number projected to rise to 783 million by 2045 [158]. Type 2 diabetes mellitus (T2DM), in particular, is closely linked to obesity, a sedentary lifestyle, and dietary factors, and it is associated with increased risks of cardiovascular disease, nephropathy, retinopathy, and neuropathy [159].

A growing body of evidence implicates oxidative stress and chronic low-grade inflammation in the pathogenesis and progression of T2DM [160]. Hyperglycemia-induced production of reactive oxygen species (ROS) can impair pancreatic β-cell function and promote insulin resistance in peripheral tissues. As a result, dietary antioxidants have attracted attention for their potential to modulate these pathogenic pathways. Among them, carotenoids, naturally occurring pigments found in colorful fruits and vegetables, have emerged as promising candidates due to their potent antioxidant, anti-inflammatory, and immunomodulatory properties [161]. Moreover, evidence suggests that dietary carotenoids may not only reduce oxidative stress associated with hyperglycemia but also improve insulin signaling pathways [162].

Epidemiological and clinical studies suggest an inverse association between dietary carotenoid intake and the risk of developing T2DM [163,164]. Specific carotenoids, such as β-carotene, lutein, zeaxanthin, and lycopene, have been examined for their capacity to improve glycemic control, reduce oxidative stress, and enhance insulin sensitivity. Therefore, this section aims to critically examine the biochemical mechanisms and therapeutic potential of carotenoids in the management of diabetes, integrating insights from mechanistic, epidemiological, and clinical research [165].

According to the above-mentioned facts, carotenoids, such as β-carotene, lycopene, lutein, and zeaxanthin, have been shown to possess antioxidant properties that can mitigate the cellular damage associated with diabetes. In diabetic states, elevated glucose levels lead to increased generation of reactive oxygen species (ROS), contributing to β-cell dysfunction and insulin resistance [161]. Carotenoids neutralize ROS and inhibit lipid peroxidation, thereby preserving cellular function. For instance, β-carotene supplementation has been reported to improve insulin sensitivity and reduce oxidative stress markers in diabetic rodent models [166]. Moreover, in diabetic human models, a randomized, placebo-controlled crossover clinical trial [167] showed improvements in insulin metabolism, reduced insulin resistance, and increased levels of plasma nitric oxide and glutathione, indicating enhanced antioxidant capacity after six weeks of supplementation (three times daily) with β-caroten (0.05 g). In addition, a longitudinal study assessed the relationship between serum β-carotene levels at age 50 and insulin sensitivity at age 70 in non-diabetic participants. The findings revealed that higher serum β-carotene concentrations were associated with improved insulin sensitivity two decades later, suggesting a potential long-term protective effect against insulin resistance [168].

Epidemiological data further support the inverse association between carotenoid intake and diabetes risk. A cross-sectional study using NHANES data found that individuals with higher plasma levels of carotenoids exhibited better glycemic control and lower insulin resistance [164]. Similarly, lycopene has been shown to reduce HbA1c levels and enhance antioxidant enzyme activity in diabetic patients [169]. Furthermore, carotenoids may exert anti-inflammatory effects by modulating signaling pathways, such as NF-κB, and inhibiting pro-inflammatory cytokine expression, mechanisms that are increasingly implicated in the pathophysiology of insulin resistance [6]. In this line, a study by Vincent et al. [170] examined the effects of an 8-week antioxidant supplementation regimen—including β-carotene (10 mg/day), vitamin C (500 mg/day), and vitamin E (800 IU/day)—on insulin sensitivity and oxidative stress in overweight and normal-weight young adults. The results indicated a 15% reduction in HOMA-IR in overweight participants, along with significant decreases in oxidative stress markers, such as lipid hydroperoxides. Additionally, there were reductions in endothelial adhesion molecules, suggesting improved vascular function.

Despite these promising findings, challenges remain. The bioavailability of carotenoids varies significantly depending on the food matrix, preparation methods, and individual factors, such as gut microbiota and genetic polymorphisms [171]. Moreover, while observational studies provide correlations, randomized controlled trials (RCTs) investigating the direct impact of carotenoid supplementation on diabetic outcomes remain limited and show mixed results. Some RCTs report significant improvements in metabolic parameters, while others show minimal to no effects, highlighting the need for standardized methodologies and longer follow-up durations. In agreement with this, a meta-analysis by Beydoun et al. [172] reported an inverse association between total carotenoids and metabolic syndrome, with β-carotene showing the strongest association among individual carotenoids. Moreover, a recent systematic review [173] found that moderate dietary intakes of β-carotene were associated with a lower risk of T2DM, potentially through the reduction of insulin resistance. However, supplementation with β-carotene did not show a significant protective effect against T2DM in randomized controlled trials. Despite these studies indicating that dietary intake of β-carotene is associated with improved metabolic parameters, supplementation does not consistently show the same benefits. This highlights the importance of obtaining β-carotene through a balanced diet rich in fruits and vegetables.

Thus, carotenoids could offer a promising complementary approach in the management of diabetes due to their potent antioxidant and anti-inflammatory properties [162]. Evidence from both animal studies and human cohorts supports their beneficial effects on glycemic control and insulin sensitivity [164]. However, variations in study designs, bioavailability, and individual responses necessitate further investigation through well-designed clinical trials [171]. As a practical point of view for sport and nutrition professionals, integrating carotenoid-rich foods into the diet may serve as a feasible strategy to support conventional diabetes therapies and reduce the burden of diabetic complications.

## 10. Green Tea Catechins and Insulin Resistance

Green tea is a popular beverage that has gained interest from scientists because of its health benefits. Catechins are polyphenolic antioxidants that account for about 30% of the ingredients present in tea and have been reported to have anti-diabetic and anti-inflammatory effects [174,175]. Among its conjugates, epigallocatechin (EGC) accounts for the majority of catechins in green tea, and it is the one that has been more broadly studied for its properties [176]. Nevertheless, other forms, such as (−)-epicatechin, have also been shown to inhibit oxidative stress and inflammation and to regulate events in digestion that affect glucose homeostasis. Multiple studies and their reviews indicate that catechins and, particularly, EGCG have benefits with regard to insulin resistance and glycemia in the context of T2DM. Catechin intake can improve insulin sensitivity and reduce FBG both in healthy individuals and those with risk factors for T2DM [177]. In addition to anti-diabetic effects, EGCG has been reported to have anti-obesity, hypotensive, and cardiovascular comorbidity prevention properties [178,179].

Green tea catechins seem to exert their benefits through several mechanisms. Studies have shown that catechins are able to inhibit carbohydrate digestive enzymes, such as α-amylase and α-glucosidase, and decrease FBG levels in vitro and in diabetic rat models. One study [180] additionally proved that EGCG could act as an insulin-mimetic compound and increase glucose transporter GLUT4 translocation to the membrane and enhance glucose absorption in L6 skeletal muscle cells through the PI3K/AKT pathway. EGCG has been reported to have some differences in the pathways affected depending on the tissue, which might be related to stated variations in its affinity for target proteins in several organs [181]. Furthermore, catechins have also been proven to enhance insulin sensitivity and reduce its resistance when combined with coffee chlorogenic acids, as shown by a decreased homeostatic model assessment of insulin resistance index (HOMA-IR) and better postprandial insulin responses [182,183]. In addition, Xin et al. [184] showed that a complex formed by three components—hawthorn polyphenols, D-chiro-inositol (DCI), and EGCG—had synergistic hypoglycemic effects mediated by PI3K/AKT/GSK-3 in the liver of induced diabetic mice, subsequently relieving insulin resistance. Hence, catechins can significantly alleviate T2DM by improving insulin sensitivity. Catechins have also been shown to exert their anti-diabetic actions through oxidative stress relieving effects [185], mitochondrial function improving effects [186], inflammation prevention [187], and intestinal microbiota regulation [177].

Despite the increasing evidence of multiple possible targets for catechins, some of their benefits in clinical trials and the optimal doses needed to activate a response are yet to be elucidated [179]. Interestingly, a randomized placebo-controlled study indicated that participants’ fasting plasma glucose decreased after three months of daily consumption of epicatechin-enriched bread [188]. Takahashi et al. additionally showed in human adults that ingestion of catechin-rich green tea during the evening, but not in the morning, decreased postprandial glucose levels. However, as stated when speaking about other compounds, the low bioavailability of catechins is a challenge to overcome in their discussed relevance for T2DM prevention or treatment [189].

## 11. Synergistic Effects of Antioxidant Combinations

Oxidative stress, a biological state characterized by an excess of reactive oxygen species (ROS) relative to the body’s capacity to neutralize them through antioxidant defenses, has emerged as a central factor in the progression of numerous chronic and degenerative diseases. These include, but are not limited to, cardiovascular disease, type 2 diabetes, neurodegenerative disorders, such as Alzheimer’s and Parkinson’s diseases, and various forms of cancer [190]. In this line, ROS can damage cellular components, including lipids, proteins, and DNA, leading to inflammation, cellular dysfunction, and, ultimately, tissue degradation.

Antioxidants, both endogenous (e.g., superoxide dismutase, glutathione) and exogenous (e.g., vitamin C, polyphenols), play a critical role in neutralizing these reactive species and maintaining redox homeostasis [191]. While individual antioxidants have been extensively studied and utilized in both dietary and therapeutic contexts, growing evidence suggests that the efficacy of antioxidant intervention can be significantly enhanced through strategic combinations. This is due to potential synergistic effects, whereby the combined action of two or more antioxidants produces a greater biological effect than the sum of their separate actions [192].

Such synergy can arise from various mechanisms; some antioxidants can regenerate others (e.g., vitamin C restoring oxidized vitamin E), while others may act on different cellular targets or within distinct compartments of the cell [193]. Additionally, the bioavailability, stability, and pharmacokinetics of certain antioxidants can be improved when administered in combination, enhancing their overall therapeutic potential [5]. Thus, these properties make synergistic antioxidant combinations a promising frontier in nutritional application.

One of the best-documented synergistic interactions is between vitamin C (ascorbic acid) and vitamin E (α-tocopherol). Vitamin E is lipid-soluble and acts primarily within cell membranes to prevent lipid peroxidation, while vitamin C is water-soluble and circulates in the aqueous compartments of the cell and plasma. When vitamin E neutralizes a free radical, it becomes a radical itself. Vitamin C can regenerate this oxidized form of vitamin E back to its active state, thereby sustaining its antioxidant activity [194]. In this line, many studies in test tubes and animal models show clear synergistic antioxidant effects, with vitamin C and E together providing stronger protection against lipid peroxidation and oxidative stress than either alone. However, while some human studies show increased antioxidant capacity and changes in blood levels of both vitamins with supplementation, the evidence for a strong synergistic effect in reducing markers of oxidative stress is less consistent [195]. Some trials found no additional benefit from combining the vitamins compared to taking either alone [196,197].

Beyond vitamins, polyphenols—naturally occurring compounds found in fruits, vegetables, tea, and wine—are potent antioxidants that often exhibit greater efficacy when used in combination. For example, the combined use of resveratrol and quercetin has been shown to exert stronger anti-inflammatory and antioxidant effects than either compound alone. A study by Pérez-Vizcaíno and Pérez Vizcaino demonstrated that these flavonoids jointly modulate signaling pathways, such as nuclear factor κB (NF-κB) and nuclear factor erythroid-like 2 (Nrf2), more effectively, contributing to reduced oxidative stress and improved vascular function [198]. Moreover, for cancer activity, this combination can also reshape the tumor microenvironment, promoting immune activation and reducing immunosuppressive cell populations, which may enhance anti-tumor responses [199].

Synergy is also observed in combinations involving glutathione (GSH), a key intracellular antioxidant, and selenium, a trace element required for the activity of glutathione peroxidase [200]. Adequate selenium intake enhances the enzymatic activity of glutathione peroxidase, allowing for more effective detoxification of peroxides and hydroperoxides in cells. In this case, one antioxidant serves as a cofactor that boosts the functional capacity of another, underlining the importance of micronutrient balance in antioxidant therapy [201]. Although most studies have been conducted in animal models, in human models, depletion of both selenium and glutathione appears to increase susceptibility to liver injury caused by drugs and toxins, highlighting their combined importance in liver protection [202]. Furthermore, regarding the combination of selenium with other elements, a randomized controlled trial by Mazloom et al. observed that a combination of vitamins C and E together with selenium significantly improved oxidative stress biomarkers and glycemic control in patients with type 2 diabetes, suggesting that such combinations could have therapeutic value in metabolic disorders [203].

Moreover, in neurodegenerative conditions, combinations, such as melatonin with α-lipoic acid, have shown promise. Melatonin, a pineal hormone with antioxidant properties, can cross the blood–brain barrier and scavenge ROS directly, while α-lipoic acid works to regenerate endogenous antioxidants like GSH and coenzyme Q10 [204]. In this line, in vitro studies show that melatonin combined with ALA more effectively reduces markers of oxidative DNA damage than either agent alone, indicating an important synergistic effect [205].

Understanding the synergistic effects of antioxidant combinations offers promising avenues for enhancing therapeutic outcomes and preventing oxidative-stress-related diseases. While several studies affirm the benefits of such combinations, further clinical research is necessary to optimize formulations, dosages, and delivery methods. This is important because not all antioxidant combinations yield synergistic effects [206]. Thus, understanding these factors is crucial for designing effective antioxidant mixtures in foods and supplements.

## 12. Gut Microbiota and Antioxidant Interactions

The human gut microbiota comprises a complex ecosystem of trillions of microorganisms that play a fundamental role in host metabolism, immune regulation, and nutrient processing. In recent years, mounting evidence has implicated gut dysbiosis, a state of microbial imbalance, as a key contributor to the development and progression of type 2 diabetes mellitus. Dysbiosis in diabetic individuals is typically characterized by reduced microbial diversity, decreased levels of beneficial commensals, and an increase in pro-inflammatory bacteria that promote intestinal barrier dysfunction, lipopolysaccharide (LPS) leakage, and low-grade systemic inflammation [75]. Importantly, this pathological process is not unidirectional. The gut microbiota dynamically interacts with dietary and pharmacological inputs, including plant-derived antioxidants, which can both influence and be influenced by the microbial ecosystem. This bidirectional relationship offers a compelling therapeutic opportunity; by modulating the microbiota with targeted antioxidant interventions, it may be possible to reduce inflammation, improve insulin sensitivity, and restore metabolic homeostasis in individuals with type 2 diabetes mellitus [207].

Plant antioxidants, such as polyphenols, flavonoids, anthocyanins, and tannins, are often poorly absorbed in the upper gastrointestinal tract. A significant proportion, estimated at over 90%, reaches the colon intact, where it encounters a dense and metabolically active microbial community. These compounds serve as substrates for microbial metabolism, undergoing deglycosylation, dehydroxylation, demethylation, and ring fission reactions that produce a wide array of bioactive metabolites, including urolithins, phenolic acids, and hydroxycinnamates [208]. These microbial transformations frequently result in metabolites with enhanced bioavailability, altered pharmacokinetics, and distinct biological activity compared to the parent compound. For instance, ellagitannins from berries and pomegranates are transformed by Gordonibacter species into urolithin A, which has been shown to improve mitochondrial function, reduce oxidative stress, and enhance insulin sensitivity in preclinical models [208,209]. Conversely, many polyphenols exert prebiotic-like effects, selectively promoting the growth of beneficial bacterial taxa, such as *Akkermansia muciniphila*, *Faecalibacterium prausnitzii*, and *Bifidobacterium adolescentis*. These microbes are associated with improved glucose tolerance, reduced adiposity, and enhanced gut barrier function, supporting the hypothesis that antioxidants may act as modulators of microbial ecology [208,210].

One of the principal mechanisms through which microbiota-mediated antioxidant effects are exerted is through the generation of short-chain fatty acids (SCFAs). SCFAs, such as butyrate, acetate, and propionate, are microbial fermentation products of dietary polyphenols and fibers. These compounds act on G-protein-coupled receptors (GPR41, GPR43) in the gut epithelium and immune cells to regulate energy metabolism, improve glucose uptake, and attenuate inflammation. Butyrate serves as the primary energy source for colonic epithelial cells, maintaining tight junction integrity and reducing intestinal permeability [211]. This limits the translocation of LPS and other microbial antigens into circulation, effectively lowering metabolic endotoxemia and the associated systemic inflammatory response, which is a known contributor to insulin resistance. In addition, SCFAs and microbial metabolites of polyphenols inhibit histone deacetylases (HDACs), promoting epigenetic changes that suppress inflammatory gene expression. Collectively, these interactions position the microbiota not just as a passive receiver of antioxidants but as an active co-contributor to their metabolic and immunomodulatory benefits [211].

Anthocyanins, found in berries, grapes, and red cabbage, are extensively metabolized by gut microbes into phenolic acids with anti-diabetic effects. These metabolites have been shown to enhance gut barrier function, reduce hepatic gluconeogenesis, and modulate glucose transporters. Moreover, anthocyanins increase the abundance of butyrate-producing bacteria, contributing to improved insulin sensitivity [212]. Resveratrol modulates the gut microbiota by suppressing the growth of opportunistic pathogens like Desulfovibrio and promoting Lactobacillus and Bifidobacterium species. This contributes to reduced inflammation and improved intestinal health. In type 2 diabetes mellitus mouse models, resveratrol supplementation was associated with improved glucose tolerance and reduced weight gain, effects that were attenuated in antibiotic-treated animals, underscoring the microbiota’s mediating role. Berberine is well-known for its antimicrobial activity, but, in subclinical doses, it selectively remodels the microbiota, increasing the relative abundance of SCFA-producing and anti-inflammatory species. This has been associated with improved glycemic control, lipid profile, and insulin sensitivity in both rodents and human trials. Notably, berberine also increases GLP-1 secretion, an effect attributed in part to changes in microbial composition [213]. Catechins from green tea promote the growth of *Akkermansia muciniphila*, a mucin-degrading bacterium associated with reduced adiposity and improved insulin sensitivity. They also inhibit Clostridium species involved in endotoxin production. These microbial shifts correspond to reductions in inflammatory cytokines, insulin resistance, and hepatic lipid accumulation in animal models [214]. Curcumin is poorly absorbed but extensively modified by gut microbes into metabolites, such as tetrahydrocurcumin, which retains anti-inflammatory and antioxidant activity. Curcumin also improves the Firmicutes-to-Bacteroidetes ratio and increases SCFA production. In diabetic rats, curcumin supplementation restored microbial diversity and improved fasting glucose and insulin resistance markers [215]. Quercetin enhances the gut’s microbial diversity and promotes colonization by Lactobacillus and Bacteroides, which improve gut barrier function and reduce inflammation. Quercetin metabolites also have insulin-sensitizing properties and reduce hepatic lipid accumulation, further supporting their role in improving glucose metabolism.

Microbiota-mediated antioxidant activity contributes not only to peripheral insulin sensitivity but also to the preservation of pancreatic β-cell function. SCFAs and polyphenol-derived metabolites exert anti-apoptotic effects on β-cells, promote insulin gene expression, and reduce oxidative damage. For instance, butyrate has been shown to increase Pdx1 and Ins1 gene expression, which is essential for β-cell identity and insulin secretion. Additionally, by reducing gut-derived inflammation, microbiota-modulating antioxidants decrease the systemic cytokine burden that impairs β-cell viability and insulin biosynthesis. Several in vivo studies demonstrate that diets enriched in antioxidant polyphenols reduce islet inflammation, increase islet mass, and restore insulin secretion in diabetic animals [216].

While most microbiota–antioxidant interactions have been studied in animal models, human data are emerging. Several randomized clinical trials have demonstrated that polyphenol-rich interventions (e.g., cranberry extract, green tea catechins, resveratrol) result in measurable changes in gut microbiota composition and correlate with improvements in HbA1c, fasting glucose, and inflammatory biomarkers in patients with type 2 diabetes mellitus. Moreover, advances in metagenomic sequencing and metabolomics have facilitated the identification of polyphenol-responder phenotypes, individuals whose microbiota composition is predictive of a favorable metabolic response to specific antioxidants [207,217]. This opens the door for personalized dietary recommendations that consider microbiota profiles to optimize antioxidant efficacy. Emerging interventions also combine prebiotics (e.g., inulin, FOS) with polyphenols to create symbiotic formulations, which have shown synergistic effects on glucose control, microbiota diversity, and SCFA production. These innovations represent a shift from general dietary guidelines to precise functional nutrition in diabetes care. Despite promising findings, several challenges remain. First, there is significant interindividual variability in microbiota composition, making it difficult to generalize outcomes [207]. Second, the dose and bioavailability of antioxidants are often inconsistent across studies, and the extent to which microbial metabolism contributes to systemic effects is not always clear. Moreover, most of the current evidence stems from animal models, and while translational potential is high, robust long-term clinical trials are needed to validate microbiota-targeted antioxidant therapies in diverse populations. Finally, integrating microbiome data into clinical practice will require user-friendly analytical tools and standardized protocols for microbiota assessment and dietary personalization [218].

**Figure 1:**
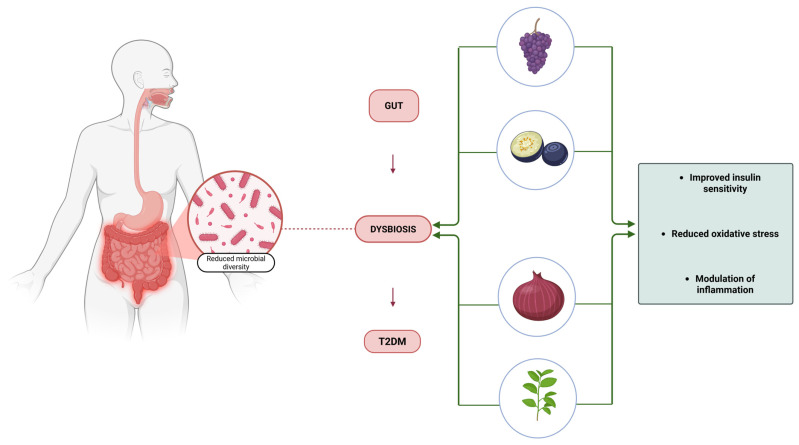
Microbiota-mediated metabolism of plant antioxidants and their impact on insulin sensitivity, oxidative stress, and inflammation.

In conclusion, plant-derived antioxidants and the gut microbiota interact in a synergistic and dynamic manner, influencing each other’s composition, bioactivity, and health effects. Through microbiota-mediated pathways, including SCFA production, metabolite transformation, gut barrier reinforcement, and immune modulation, antioxidants exert profound effects on insulin sensitivity, inflammation, and glycemic control in type 2 diabetes mellitus. This growing body of evidence underscores the potential of microbiota-aware dietary strategies and precision antioxidant therapy in diabetes management. Future research should focus on identifying microbial biomarkers of responsiveness, optimizing polyphenol delivery systems, and conducting integrative human trials to translate this promising science into practical, individualized interventions for type 2 diabetes mellitus care (Figure 1).

## 13. Nutrigenomics and Personalized Antioxidant Therapy

The interaction between nutrition and genetic factors has long been suggested and, in some cases, directly linked to specific diseases. Research over the years trying to understand this connection has referred to it as *nutrigenomic*, and now it is recognized as a key contributor to the development and progression of various health conditions [219]. Nutrigenomics proposes that the body possesses a distinct signaling system that predisposes individuals to specific patterns of gene expression. In this context, nutrients consumed act as stimuli that are detected by cellular sensory mechanisms, subsequently influencing the expression of genes, proteins, and metabolites. Moreover, nutrigenomics seeks to elucidate how nutrition contributes to maintaining physiological homeostasis and to identify cellular interactions that activate inflammatory stress pathways, thereby enhancing our understanding of diet-related diseases [220]. This discipline highlights the dynamic interplay between bioactive dietary compounds and genetic activity. Additionally, it incorporates approaches from nutritional systems biology to identify biomarkers that indicate susceptibility to nutrition-associated pathologies [221]. Nutrigenetics examines how individual genetic variations affect responses to specific nutrients, influencing dietary needs and disease risk. In contrast, nutrigenomics explores how nutrients and bioactive food compounds regulate gene expression and cellular processes [222]. Together, they form the basis of personalized nutrition and the prevention of diet-related diseases.

### 13.1. Nutrigenomics and the Role of the Microbiome

At the core of this discipline lies the concept that dietary components serve not only as sources of energy and structural elements but also as signaling molecules capable of modulating transcriptional activity. These nutrient–gene interactions impact key physiological processes, including inflammatory responses, oxidative stress regulation, and metabolic homeostasis [219]. Understanding these mechanisms is essential for identifying the molecular pathways involved in diet-related diseases and developing targeted nutritional strategies aimed at disease prevention and health optimization.

The gut microbiome is crucial in modulating gene expression, metabolism, and immune responses. The human microbiome, composed of approximately 40 trillion microorganisms, interacts dynamically with dietary components, influencing health outcomes through complex molecular pathways [223]. Alterations in microbiota composition—due to infection, antibiotics, lifestyle, or diet—can shift the balance toward pro-inflammatory or protective profiles [45]. For instance, specific microbial species, such as *Veillonella* and *Streptococcus*, have been detected in atherosclerotic plaques, while *Akkermansia muciniphila* has shown protective effects against diet-induced atherosclerosis [224]. Also, the balance between bacterial families like *Firmicutes* and *Bacteroidetes* has also been linked to obesity risk [225,226]. Probiotics, which include strains like *Lactobacillus* and *Bifidobacterium*, exert beneficial effects on gastrointestinal health, lactose intolerance, and, possibly, metabolic and bone disorders [225]. Novel approaches, such as fecal microbiota transplantation, further illustrate the therapeutic potential of modulating microbial populations [227].

Beyond the influence of the microbiome, the molecular basis of nutrigenomics also involves a range of metabolic enzymes whose activity is modulated by specific dietary components. These enzymes play a pivotal role in processing bioactive compounds, mediating gene–nutrient interactions, and, ultimately, shaping individual susceptibility to disease [228]. Key enzymes, such as Cytochrome P450 (CYPs), are involved in the oxidation of various dietary compounds; for example, the activity of CYP1A2 can be induced by indole-3-carbinol from cruciferous vegetables or inhibited by naringenin in grapefruit [135], modulating the metabolism of potential carcinogens [229,230]. Similarly, Glutathione S-transferases (GSTs)—especially GSTM, GSTP, and GSTA isotypes—serve as detoxifying enzymes by conjugating reduced glutathione to reactive electrophiles, thereby preventing DNA damage and mutagenesis [231]. Impaired GST activity has been associated with increased disease susceptibility. Additionally, as specified by Mishra et al., MTHFR (methylenetetrahydrofolate reductase) plays a central role in one-carbon metabolism, with the 677C→T polymorphism affecting enzyme efficiency and influencing folate requirements and disease risk. For instance, individuals with the TT genotype may require higher folate intake to mitigate risks of vascular and neoplastic diseases [232]. These interactions underscore the bidirectional relationship between diet and gene expression mediated by microbial metabolites, revealing promising strategies for personalized nutrition and antioxidant therapy. Incorporating microbiome dynamics into nutrigenomic models may enhance the precision and efficacy of dietary interventions aimed at preventing chronic diseases [219,220].

### 13.2. Genetic Variability and Personalized Antioxidant Strategies

Research has further demonstrated that the diet–disease relationship is modulated by genetic and ethnic variability. For instance, one study found a significantly higher risk of disease among Sudanese individuals with the glutathione S-transferase M1 null genotype who consumed aflatoxin-contaminated peanut butter compared to those without this genetic variant [233]. Ongoing research continues to elucidate the mechanisms through which genetic makeup determines the absorption, metabolism, and excretion of nutrients, as well as how specific nutrients modulate gene expression. For instance, previous authors highlighted how genetic factors influence blood lipid profiles and cardiovascular risk, offering valuable insights for personalized dietary prevention strategies. This highlights that understanding these gene–diet interactions is key to advancing precision nutrition and developing targeted interventions for chronic disease prevention [234].

In this regard, a recent study investigated the effects of a dietary intervention based on resveratrol, green tea extract, α-tocopherol, vitamin C, n−3 (omega-3) polyunsaturated fatty acids, and tomato extract. It was discovered that a diet against modulated inflammatory processes and oxidative stress markers may be a potential targeted strategy in managing inflammation-related conditions [235]. Concretely, the study demonstrates that a combination of compounds with antioxidant properties can favorably modulate markers of inflammation and oxidative stress [236]. For instance, a meta-analysis examining the effects of antioxidant therapy on chronic kidney disease (CKD) progression found that despite heterogeneity among studies, antioxidant therapy appeared to reduce CKD progression. Specifically, compounds like pentoxifylline and bardoxolone methyl demonstrated robust and statistically significant protective effects, highlighting the potential of personalized antioxidant interventions in managing CKD [237]. In another example, a randomized clinical trial investigated the impact of mixed apple and bergamot juice (MAB juice) supplementation on oxidative stress and inflammation. Over a two-week period, 24 subjects received MAB juice supplementation, resulting in positive effects on body composition, biochemical profiles, and the expression of oxidative and inflammatory genes. The study underscores the potential of personalized dietary interventions rich in antioxidants to modulate gene expression and improve health outcomes [238].

For instance, a study explored the potential of dihydromyricetin (DHY), a flavonoid with potent antioxidative properties, in managing diabetic cardiomyopathy. The research demonstrated that DHY significantly enhanced cardiac function and reduced myocardial injury by activating Sirtuin 3 (SIRT3), a mitochondrial protein involved in cellular stress responses, thereby offering a promising therapeutic avenue for cardiovascular complications associated with diabetes [239]. In another example, a systematic review assessed the efficacy of antioxidant therapy in enhancing the quality of life of patients with chronic pancreatitis (CP). The findings suggested that antioxidant therapy holds potential in symptom management; however, the results were mixed, indicating the necessity of more rigorous, larger-scale studies to confirm its effectiveness and establish standardized treatment protocols [240]. Furthermore, research into the nutrigenetics of antioxidant enzymes has highlighted how genetic variations can influence individual responses to oxidative stress and viral infections [241].

Understanding these genetic differences is crucial for developing personalized antioxidant strategies that effectively modulate oxidative stress and enhance immune responses. These studies collectively emphasize the importance of personalized antioxidant strategies in managing and potentially mitigating various health conditions by considering individual genetic and biochemical profiles.

## 14. Plant-Derived Antioxidants in Young Adults, Older Adults, and Pregnant Women with Diabetes

Beyond general mechanisms and pharmacokinetics, antioxidant interventions must also be adapted to the unique physiological conditions of special populations, such as pregnant women, older adults, and individuals with comorbidities. As known, the burden of diabetes is increasing globally across all age groups, with oxidative stress playing a central role in its pathogenesis and complications. Plant-derived antioxidants have emerged as promising nutritional tools to counteract oxidative damage and improve glycemic control.

### 14.1. Plant-Derived Antioxidants in Young Adults with Diabetes

In young adults with type 1 or type 2 diabetes, dietary antioxidants may positively influence glycemic control and reduce oxidative stress. Polyphenols—bioactive compounds found in fruits, vegetables, and whole grains—act as potent antioxidants and exhibit anti-inflammatory properties, helping to prevent chronic conditions associated with diabetes [242]. Furthermore, studies have shown that antioxidant-rich diets are inversely associated with oxidative-stress-induced conditions, such as insulin resistance, a key factor in the development of type 2 diabetes [243]. Regarding this, Gutierrez et al. developed a study involving sedentary, obese young women (average age of 22.7 years) to assess the effects of consuming 5 g of encapsulated Cassia cinnamon bark daily. The results indicated a significant 10.1% reduction in blood glucose levels and improved glucose tolerance compared to a placebo group, highlighting cinnamon’s potential in managing blood sugar levels in young adults with diabetes [244]. However, there was no improvement in insulin resistance in young, sedentary, obese women. Another study focused on the impact of 12-week ubiquinone (coenzyme Q10) supplementation in well-trained college athletes. Ho et al. pointed out that higher ubiquinone status was associated with improved antioxidant capacity and glycemic control, highlighting its potential role in managing blood sugar levels among physically active individuals [245].

Another study focused on resveratrol administration pointed out that 800 mg/day for two months to patients with type 2 diabetes mellitus (T2DM) resulted in an 8% reduction in malondialdehyde (MDA) levels and an 18.54% decrease in carbonyl protein, markers of oxidative stress. Additionally, total thiol levels increased by 12%, nitric oxide synthase (NOS) by 3%, and catalase by 12%, indicating enhanced antioxidant defenses [246]. Also, in patients with a history of 3.5 years, daily supplementation of 3 g of L-citrulline for two months resulted in a 16% reduction in fasting blood glucose levels and a 25% decrease in MDA levels. Moreover, there were significant increases in serum levels of nitric oxide (27%), superoxide dismutase (2%), and glutathione peroxidase (2.2%), suggesting improved oxidative stress markers [246]. Montonen et al. also specified that a higher dietary intake of vitamin E was significantly associated with a reduced risk of developing type 2 diabetes. Specifically, the relative risk (RR) of type 2 diabetes between the highest and lowest quartiles of vitamin E intake was 0.69, indicating a potential protective effect of this antioxidant vitamin [247].

Nevertheless, not all studies have yielded positive outcomes. A systematic review of medicinal plants used for diabetes treatment found that among the studies reviewed, only the trial involving bitter melon did not show any significant change in blood glucose levels after intervention. This underscores the importance of rigorous clinical evaluation to validate the efficacy of plant-derived antioxidants in diabetes management [248]. However, findings highlight the potential of plant-derived antioxidants in managing diabetes among young adults, while also emphasizing the need for further research to confirm their efficacy and safety.

### 14.2. Plant-Derived Antioxidants in Older Adults with Diabetes

Aging is associated with increased oxidative stress and reduced endogenous antioxidant capacity, exacerbating diabetic complications in older adults. Diets high in antioxidant-rich foods, such as nuts, have demonstrated notable health benefits [249,250]. For example, walnut consumption has been linked to improved cognitive performance in young adults, while pistachios, rich in antioxidants, may enhance eye health [251]. Additionally, Asp et al. demonstrated that safflower oil, abundant in unsaturated fatty acids and antioxidants, may improve blood glucose control, particularly in postmenopausal women with diabetes [252]. A randomized clinical trial evaluated the effects of resveratrol (RV) supplementation on oxidative stress markers and sirtuin 1 levels in older adults with type 2 diabetes. Ninety-seven participants received either 1000 mg/day or 500 mg/day of RV, or a placebo, over six months. The study found that RV supplementation positively influenced oxidative stress markers and sirtuin 1 levels, suggesting benefits for older adults managing diabetes [253]. Appiah et al.’s research investigated the impact of Bridelia ferruginea tea on antioxidant status in individuals with type 2 diabetes. The findings revealed that participants who consumed the tea exhibited significantly higher antioxidant levels compared to those who did not, indicating its potential to enhance antioxidant defenses in older adults with diabetes [254].

Additionally, Garcia Martínez and collaborators showed in a randomized controlled trial the impact of resveratrol supplementation on oxidative stress markers and sirtuin 1 levels in older adults with type 2 diabetes. Concretely, ninety-seven participants received either 1000 mg/day or 500 mg/day of resveratrol, or a placebo, over six months. The study found that resveratrol supplementation positively influenced oxidative stress markers and sirtuin 1 levels, suggesting benefits for older adults managing diabetes [253]. Furthermore, a study on dietary antioxidant intake and risk of type 2 diabetes observed that higher intake of antioxidants, such as vitamin E and β-cryptoxanthin, was associated with a reduced risk of developing type 2 diabetes, highlighting the importance of antioxidant-rich diets in older adults [247,255].

Taken together, these findings support the potential of plant-derived antioxidants as a complementary strategy to enhance metabolic control, reduce oxidative stress, and mitigate diabetes-related complications in the aging population.

### 14.3. Plant-Derived Antioxidants in Pregnant Women with Diabetes

**Figure 2:**
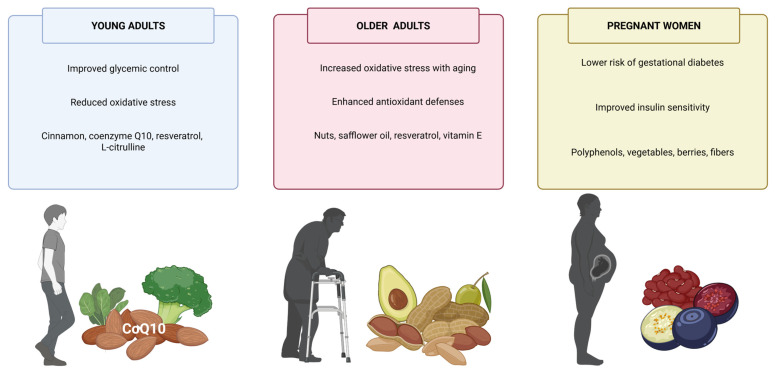
Plant-derived antioxidants in young adults, older adults, and pregnant women with diabetes.

Gestational diabetes (GDM) is a common pregnancy complication with health risks for both mother and child. Emerging evidence suggests that antioxidant-rich diets during early pregnancy are associated with a lower risk of developing GDM [256]. A recent study carried out by Heshmati and collaborators found that higher dietary total antioxidant capacity (DTAC) in early gestation significantly reduced GDM risk, highlighting the protective role of dietary antioxidants [257]. Moreover, increased intake of vegetables, fibers, and fruits has been shown to lower inflammation by boosting antioxidant levels, thereby improving insulin sensitivity and overall metabolic control [258]. Additionally, a meta-analysis explored the association between polyphenol consumption and the risk of GDM and preeclampsia (PE). While the overall findings were inconclusive, the study noted that total polyphenol intake was associated with a lower likelihood of developing GDM, indicating the potential of personalized polyphenol-rich dietary interventions in GDM prevention [259]. Moreover, research has identified several health benefits of bioactive phytochemicals, including antioxidant and anti-inflammatory activities, as well as normalizing glucose metabolism. Dietary fruits, such as acai, goji berries, blueberries, and strawberries, have high levels of antioxidants, fibers, vitamins, minerals, and phytochemicals, which have been associated with decreased risk of diabetes [260]. Furthermore, a meta-analysis examining polyphenol-rich food consumption during pregnancy found that while overall polyphenol intake did not show a significant association with GDM risk, higher total polyphenol intake was linked to a lower likelihood of developing GDM [253]. This suggests that specific polyphenol-rich foods may offer protective benefits against GDM. Additionally, Chen at al. in their study highlighted that adherence to a healthful plant-based diet before pregnancy is associated with a lower risk of GDM. This underscores the potential of plant-based dietary patterns in reducing GDM risk [261]. Collectively, these studies highlight the potential benefits of incorporating antioxidant-rich, plant-based foods into the diets of pregnant women to mitigate the risk of GDM (Figure 2).

## 15. Challenges in Bioavailability and Stability

Despite their promising biological properties, the clinical effectiveness of plant-derived antioxidants in the management of T2DM remains significantly limited by poor bioavailability, low solubility, instability in physiological environments, and extensive metabolism in the gastrointestinal tract and the liver [259]. These limitations reduce the proportion of active compounds reaching target tissues at therapeutic concentrations and compromise their potential to exert glycemic or antioxidative effects in vivo [260]. Flavonoids, such as quercetin and catechins, as well as polyphenols, like curcumin and resveratrol, illustrate this challenge. Although they display potent antioxidant, anti-inflammatory, and insulin-sensitizing actions in vitro and in animal models, their oral bioavailability in humans is typically less than 2% due to rapid metabolism, poor intestinal absorption, and first-pass hepatic clearance [261,262]. Curcumin, in particular, undergoes rapid glucuronidation and sulfation, which severely limit its systemic availability [263]. Quercetin is similarly subject to extensive conjugation, with only trace amounts of the aglycone form detectable in circulation after ingestion [264].

Furthermore, dietary interactions may exacerbate these bioavailability issues. Polyphenols can form insoluble complexes with dietary proteins or fiber, reducing their intestinal uptake. Environmental and processing factors, such as pH, light, oxygen exposure, and temperature, also compromise compound stability, both during food preparation and gastrointestinal digestion [265]. Another layer of complexity is introduced by interindividual variability in gut microbiota composition. Gut microbes play a crucial role in metabolizing polyphenols into smaller and often more bioactive derivatives—for instance, ellagitannins into urolithins or daidzein into equol. However, not all individuals possess the microbial species required for these conversions, leading to variable therapeutic responses [266]. This metabolic interdependence between host and microbiota underscores the importance of personalized approaches when considering polyphenol-based interventions for T2DM.

To address these limitations, various formulation strategies have been developed to enhance the stability and bioefficacy of antioxidant compounds. Nanoparticle carriers, liposomes, phospholipid complexes (e.g., phytosomes), and co-administration with bioenhancers, such as piperine, have shown promise in preclinical and clinical settings [267,268]. These technologies aim to protect sensitive compounds from degradation, improve intestinal permeability, prolong systemic circulation time, and facilitate targeted delivery to tissues of interest. For example, piperine has been demonstrated to increase curcumin’s bioavailability by up to 2000% by inhibiting hepatic and intestinal glucuronidation [269]. Despite these advances, clinical evidence remains limited and inconsistent. Few studies have systematically evaluated the pharmacokinetics of improved formulations in diabetic populations, and standardization of protocols remains a challenge. Additionally, regulatory hurdles and manufacturing scalability present barriers to the widespread adoption of these advanced delivery systems.

Overcoming the challenges of poor bioavailability and instability is critical for realizing the full therapeutic potential of plant antioxidants in T2DM. Without addressing these pharmacokinetic and physicochemical limitations, even compounds with strong in vitro efficacy are unlikely to translate into clinically meaningful outcomes. Future research should prioritize robust pharmacokinetic modeling, including absorption, distribution, metabolism, and excretion studies, to better understand the behavior of these compounds in the human body. Additionally, stratification of patient subgroups based on gut microbiota composition and metabolic phenotype could identify responders and non-responders, enabling precision-targeted interventions. The development of scalable, standardized delivery systems, such as nanoemulsions, polymeric micelles, or bioadhesive hydrogels, represents a promising strategy to improve intestinal absorption and metabolic stability while ensuring reproducibility and regulatory compliance. These systems must also demonstrate safety, biocompatibility, and cost-effectiveness for eventual translation into nutraceutical or pharmaceutical applications. Moreover, integrating nutrigenomics and metabolomics with clinical phenotyping can deepen our understanding of how genetic variations and metabolic status modulate antioxidant efficacy. This systems biology approach may allow for the formulation of personalized nutrition strategies that align antioxidant selection and dosing with individual genetic and microbial profiles, thereby optimizing therapeutic outcomes in T2DM. Ultimately, a multidisciplinary framework combining molecular nutrition, pharmaceutical technology, microbiome science, and clinical pharmacology will be essential to bridge the current gap between mechanistic insights and real-world effectiveness of plant-derived antioxidants in metabolic disease management.

## 16. Future Directions in Functional Foods and Therapeutics

The future of T2DM management increasingly points toward the use of functional foods and therapeutic formulations enriched with plant-derived antioxidants. These interventions aim not only to provide nutritional value but to actively modulate key metabolic dysfunctions associated with hyperglycemia, insulin resistance, and chronic oxidative stress [270]. A key area of innovation involves the integration of advanced delivery systems to overcome the poor bioavailability of polyphenols. Nanoencapsulation, liposomal carriers, and biopolymer-based matrices have demonstrated improved gastrointestinal stability and absorption of antioxidants like curcumin, resveratrol, and epigallocatechin gallate [271,272]. For example, nanoemulsified curcumin has been shown to increase plasma levels and enhance glucose uptake in diabetic models [273].

In parallel, the synergistic combination of antioxidants with dietary fibers, probiotics, or omega-3 fatty acids in food matrices is being explored. These combinations may potentiate metabolic effects by acting on multiple targets and by promoting the gut microbiota’s ability to biotransform polyphenols into bioactive metabolites [274]. Indeed, functional foods combining anthocyanins with prebiotic fibers have shown enhanced effects on insulin sensitivity and microbiota diversity in individuals with impaired glucose metabolism [275]. The emergence of precision nutrition and personalized therapeutic approaches is also shaping future strategies. Interindividual differences in gut microbiota composition, genetic polymorphisms (e.g., in SIRT1, Nrf2, or GST genes), and metabolic profiles influence responsiveness to antioxidant interventions [276]. Several clinical studies have demonstrated that individuals with specific gut microbiota enterotypes respond more favorably to polyphenol-rich diets in terms of glycemic and lipid control [277].

Furthermore, regulatory harmonization and clinical validation will be necessary to legitimize functional foods as therapeutic tools. Currently, most polyphenol-enriched products fall under nutraceutical or food supplement categories, limiting their approved health claims. Rigorous randomized controlled trials, standardized biomarkers (e.g., HbA1c, HOMA-IR, 8-isoprostanes), and robust manufacturing protocols are needed to support clinical applications [278]. Lastly, long-term cohort and intervention studies must be prioritized to determine sustained efficacy, optimal dosing, and potential interactions with standard pharmacological treatments. While many clinical trials report short-term improvements in oxidative markers and insulin sensitivity, data on long-term outcomes, such as diabetes remission, cardiovascular risk reduction, or complication prevention, remain scarce [279].

Several strategic directions are currently being explored to enhance the efficacy, applicability, and personalization of antioxidant-based functional foods in T2DM. These include the application of nanotechnology for compound delivery, microbiota-responsive formulations, regulatory standardization for therapeutic claims, and the integration of nutrigenomic tools for individualized interventions. A summary of these key innovations, their descriptions, expected benefits, and supporting references is provided in Table 2, which offers a consolidated overview of the current roadmap guiding future development in this field.

The evolution of functional foods and therapeutics for T2DM hinges on the convergence of food technology, precision medicine, microbiome science, and regulatory support. These interdisciplinary pillars provide the foundation for translating promising bioactive compounds from bench to bedside. Food technology offers tools for enhancing stability, sensory integration, and bioavailability of antioxidant compounds through advanced encapsulation, emulsification, and matrix engineering. Simultaneously, precision medicine enables the stratification of individuals based on genetic, metabolic, and microbial profiles, ensuring that interventions are tailored to those most likely to benefit. Moreover, the role of the gut microbiota is increasingly recognized as a key mediator of polyphenol metabolism and systemic efficacy, prompting the need to incorporate microbiome-responsive formulations and prebiotic–antioxidant synergies into product design. Regulatory frameworks must also adapt to the growing scientific evidence supporting the therapeutic utility of functional foods, providing clear pathways for claim substantiation, quality control, and post-market surveillance.

Developing tailored, bioavailable, and clinically validated antioxidant-based products represents a promising strategy to complement conventional diabetes management, reduce treatment burden, and delay or prevent disease progression. Ultimately, such interventions may fill a critical gap between dietary guidance and pharmacological therapy, offering scalable, sustainable, and patient-centered solutions in the long-term care of T2DM. The integration of these approaches not only enhances therapeutic precision but also aligns with public health goals aimed at reducing the global burden of metabolic diseases through preventive and lifestyle-based strategies.

## 17. Ethical Considerations and Regulatory Implications

As plant-derived antioxidant strategies progress from experimental to clinical and public health applications, ethical and regulatory frameworks must evolve in parallel. Key considerations include the following.

- Safety and efficacy standards: Nutraceuticals and functional-food-based interventions must undergo rigorous testing to demonstrate not only efficacy but also long-term safety, particularly when targeting vulnerable populations with metabolic disorders.
- Informed consent and transparency: In precision nutrition approaches involving omics data, individuals must be fully informed about how their biological data will be used, stored, and interpreted.
- Equitable access: There is a risk that personalized interventions (e.g., microbiome profiling, metabolomic-guided nutrition) may be available only to higher-income populations. Ethical implementation should ensure these innovations do not widen health disparities.
- Data privacy and autonomy: Especially relevant in digital health tools, strict standards must be applied to protect personal health data and guarantee user autonomy over dietary or therapeutic recommendations.
- Regulatory harmonization: Coordination between food, medical, and digital health regulatory bodies is essential to establish consistent approval pathways and avoid gaps in oversight as these hybrid interventions enter the market.

## 18. Conclusions

Plant-derived antioxidants offer a multifaceted approach to the prevention and management of type 2 diabetes mellitus (T2DM) by acting through mechanisms that target oxidative stress, inflammation, and insulin resistance. This review has highlighted not only their molecular and clinical relevance but also key translational challenges, including bioavailability limitations, formulation technologies, and microbiome interactions. Moreover, special populations, such as pregnant women and older adults, present unique physiological contexts that must be considered in the design of antioxidant-based interventions. The integration of personalized nutrition, advanced delivery systems, and microbiome-targeted strategies holds promise for enhancing the therapeutic impact of these compounds. Future research should focus on long-term clinical validation and regulatory standardization to enable the safe and effective implementation of antioxidant therapies in diverse populations. An interdisciplinary approach will be essential to translate the potential of plant-derived antioxidants into practical, patient-centered tools for T2DM care.

In this context, it is essential to consider not only the efficacy of these strategies but also their developmental maturity and realistic timelines for integration into clinical and dietary practice. The following Table 3 summarizes the current translational status and estimated time to practical application for several of the key innovations discussed in this review.

## Figures and Tables

**Table 1:** Comparative Overview of Plant-Derived Antioxidants in T2DM Management.

Antioxidant Group	Representative Compounds	Primary Molecular Targets	Mechanisms of Action	Clinical Evidence	Key Limitations	Potential Applications
Polyphenols	Quercetin, Resveratrol, EGCG	IRS/PI3K/Akt, NF-κB, AMPK, Nrf2	↑ Insulin sensitivity, ↓ inflammation, ↑ antioxidant defense	Strong (RCTs and meta-analyses)	Poor bioavailability; metabolism-dependent effects	Adjunct therapy, personalized nutrition
Curcuminoids	Curcumin	NF-κB, JNK, AMPK, PPARγ	Anti-inflammatory, antioxidant; modulates insulin signaling	Moderate (human studies, meta-analyses)	Low solubility; variable absorption	Formulated supplements, nano-delivery systems
Carotenoids	β-Carotene, Lutein	Nrf2, mitochondrial ROS	ROS scavenging, ↓ lipid peroxidation, ↓ AGEs	Limited but promising	Lipophilicity; food matrix dependent	Ocular protection, vascular support
Vitamins	Vitamin C, Vitamin E	Nrf2, NF-κB, eNOS	Redox balance, ↑ NO, ↓ systemic inflammation	Mixed results, dose-dependent	Variable efficacy; threshold effects	Complementary antioxidant support
Lignans and Stilbenes	Secoisolariciresinol, Resveratrol	SIRT1, PGC-1α, NLRP3	↑ Mitochondrial biogenesis, ↓ inflammasome activation	Emerging evidence	Low bioavailability; population-specific response	Gut microbiota modulation, metabolic flexibility

**Table 2:** Strategic directions for functional foods and antioxidant therapeutics in T2DM.

Strategy/Innovation	Description	Expected Benefit	Reference
Nanoencapsulation of polyphenols	Use of liposomes, nanoemulsions, or solid lipid nanoparticles for oral delivery	Improved bioavailability and stability of antioxidants	[271,272]
Functional food matrices with synergistic components	Formulation of polyphenols with fibers, omega-3, or probiotics (e.g., synbiotics)	Enhanced metabolic action and gut microbiota modulation	[274,275]
Personalized antioxidant therapy	Tailoring interventions based on genetics, gut microbiota, and metabolic phenotype	Improved individual response and efficacy	[276,277]
Regulatory standardization and clinical validation	Harmonized biomarkers, health claims approval, and randomized controlled trials (RCTs)	Therapeutic legitimacy and broader clinical integration	[278]
Integration with pharmacologic therapy	Co-administration of functional foods with drugs like metformin	Potential for synergistic glycemic and oxidative benefits	[272]
Long-term cohort and intervention studies	Evaluation of functional food efficacy over months/years in diverse populations	Insight into sustainability, adherence, and real-world outcomes	[279]
Precision nutrition and nutrigenomics application	Genotype- and microbiome-driven customization of dietary antioxidant strategies	Optimal antioxidant selection and dose per patient	[276]
Combination of multiple polyphenols with complementary targets	Multi-compound formulations targeting inflammation, oxidative stress, and insulin signaling	Amplified metabolic impact through multitarget modulation	[270,273]

**Table 3:** Practical relevance and expected implementation horizons.

Strategy/Innovation	Current Development Stage	Estimated Time to Broad Clinical/Practical Use	Expected Implementation Horizon	Notes
Microbiome-targeted therapies	Early-stage clinical trials; functional food applications	3–5 years	*Short- to mid-term*	Includes polyphenol–microbiota interaction studies; high translational potential
Precision nutrition (nutrigenomics, metabolomics)	Pilot programs and academic research	5–10 years	*Mid- to long-term*	Requires omics integration, AI tools, and regulatory support
Nanoencapsulation of antioxidants	Preclinical and emerging commercial products	3–5 years	*Short- to mid-term*	Focused on enhancing bioavailability and compound stability
Synergistic antioxidant combinations	Product formulation and validation in progress	2–4 years	*Short-term*	May reach market rapidly via nutraceutical and food industry channels
Digital health integration for antioxidant-based interventions	Conceptual and pilot phases	5–8 years	*Mid-term*	Dependent on digital platforms, mobile tech, and personalization tools

## Data Availability

Not applicable.
